# Rational design of block copolymer self-assemblies in photodynamic therapy

**DOI:** 10.3762/bjnano.11.15

**Published:** 2020-01-15

**Authors:** Maxime Demazeau, Laure Gibot, Anne-Françoise Mingotaud, Patricia Vicendo, Clément Roux, Barbara Lonetti

**Affiliations:** 1 Laboratoire des IMRCP, Université de Toulouse, CNRS UMR 5623, Université Toulouse III - Paul Sabatier, 118 route de Narbonne, 31062, Toulouse, France

**Keywords:** intracellular targeting, micelles, photodynamic therapy (PDT), photochemistry, polymer, self-assembly

## Abstract

Photodynamic therapy is a technique already used in ophthalmology or oncology. It is based on the local production of reactive oxygen species through an energy transfer from an excited photosensitizer to oxygen present in the biological tissue. This review first presents an update, mainly covering the last five years, regarding the block copolymers used as nanovectors for the delivery of the photosensitizer. In particular, we describe the chemical nature and structure of the block copolymers showing a very large range of existing systems, spanning from natural polymers such as proteins or polysaccharides to synthetic ones such as polyesters or polyacrylates. A second part focuses on important parameters for their design and the improvement of their efficiency. Finally, particular attention has been paid to the question of nanocarrier internalization and interaction with membranes (both biomimetic and cellular), and the importance of intracellular targeting has been addressed.

## Review

### Introduction

After Paul Ehrlich, in 1900, had the very first notion of a drug being delivered at will to a specific site [1], researchers have been elaborating different strategies to achieve this goal. The discovery made by Matsumura and Maeda in the late 1990’s that some macromolecular therapeutics spontaneously accumulate in inflamed or cancerous tissues, the so-called enhanced permeability and retention (EPR) effect [2–3], constituted the triggering factor for the development of a whole new part of medicine, namely nanomedicine. Indeed, the observed spontaneous accumulation was explained by the existence of disjunctions between endothelial cells in the proximity of inflamed and cancerous tissues, which enable entities smaller than these gaps to leave the bloodstream. Secondly, defects of the lymphatic system in tumors prevent these macromolecular therapeutics to be cleared from the tumor, giving them additional time to release their active cargo. This was the first example of targeting tumor tissues, exploiting only the size of the therapeutics, and is usually referred to as passive targeting. At that time, researchers got on the lead to develop intravenous nanocarriers of appropriate size (typically 20–200 nm) to benefit from this EPR effect without being cleared too rapidly through kidneys [4]. This implied a required blood circulation time of at least 24–48 h, which is the time necessary for the EPR effect to occur [5]. However, the first nanocarriers were observed to be rapidly cleared from the body or accumulated in the liver or the spleen [4]. The reason was that they were detected as foreign bodies and taken care of by opsonins, leading to their handling by the mononuclear phagocyte system. Carriers avoiding detection by opsonins had then to be developed and very common polymers, namely poly(ethylene glycol) (PEG) or poly(ethylene oxide) (PEO), were found to fulfil this requirement [6]. In parallel to this development of stealth nanocarriers, polymer chemistry had progressed strongly with the emergence of controlled polymerization. After the discovery of so-called living polymerization (polymerization without any transfer nor any termination reaction) in the 1950’s, the development of controlled radical polymerization in the 1990’s provided polymer scientists with a range of chemical tools to synthesize polymers and copolymers exhibiting various architectures, from block and gradient to grafted polymers [7]. Designing new nanocarriers exhibiting an external shell based on PEG and a core that could be either hydrophobic or a polyelectrolyte enabled the creation of numerous systems [8]. Depending on their structure, copolymers may also form self-assemblies. This is typically the case for amphiphilic block copolymers, which can form in aqueous solution polymer nanoobjects such as micelles or vesicles. The driving forces of this assembly are a loss of entropy during the self-assembly and different interactions acting on the monomer units of the polymer. Whereas polymer/polymer interactions are favored for the hydrophobic block, interactions between the hydrophobic block and water are strongly disfavored, leading to the isolation of the hydrophobic block into core or membranes [9]. In the last twenty years, thousands of papers have been published on this topic and the reader is referred to recent reviews [10–14]. Basically, the desired properties of an ideal intravenous polymer nanocarrier are biocompatibility, stealthiness, optimal size (20–200 nm), polymer/drug affinity compatible with good encapsulation and release, and a design compatible with the targeted organ [4] (this includes the possible crossing of biological barriers).

**Figure 1:**
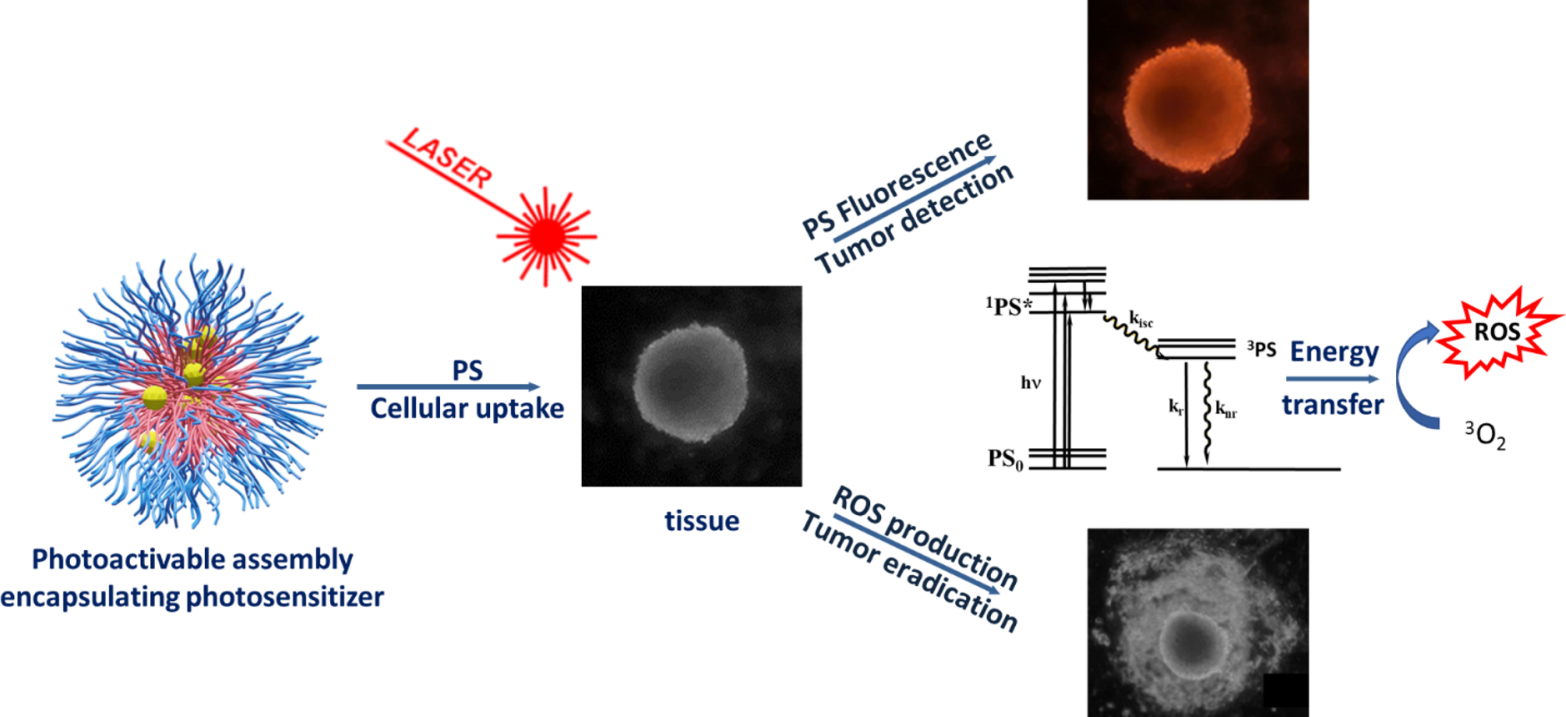
Schematic description of in vitro PDT processes using photosensitizer (PS) encapsulated in a block copolymer self-assembly. ROS: reactive oxygen species.

**Figure 2:**
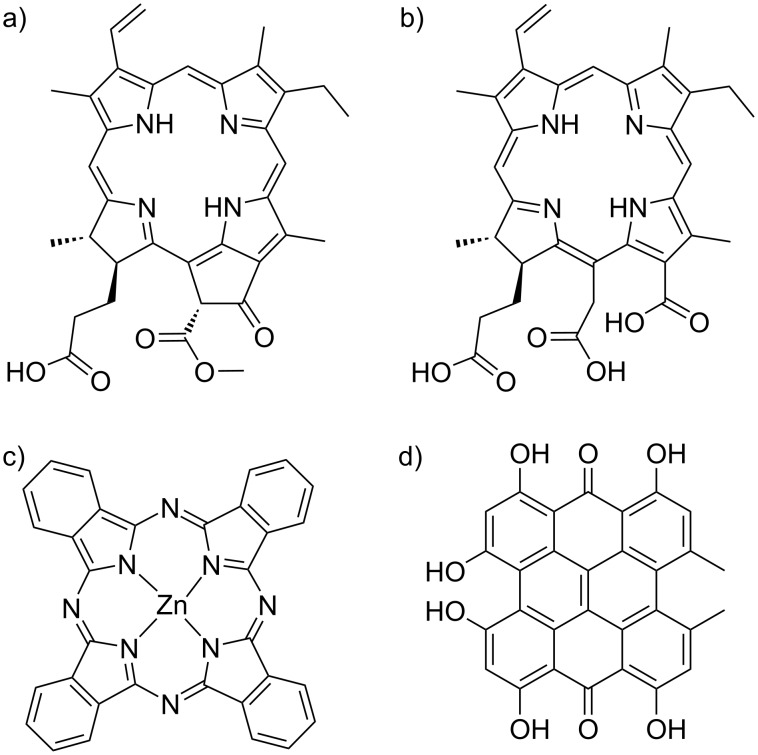
Chemical structures of four molecular photosensitizers commonly used: a) pheophorbide a; b) chlorin-e6; c) zinc phthalocyanine; d) hypericin.

The aim of this review is to focus on the benefits provided by block copolymers in photodynamic therapy (PDT), as described schematically in Figure 1 [15]. Its concept lies in the use of photosensitizing molecules that have the ability to transfer their energy to oxygen upon irradiation, leading to the in situ formation of reactive oxygen species (ROS) and the subsequent killing of the surrounding biological tissue. Photosensitizers are chosen to absorb efficiently in the 600–800 nm range in the so-called phototherapeutic window, where biological components have minimal absortion [16]. Photosensitizers are either small molecules exhibiting polycyclic structures such as porphyrins (Figure 2), inorganic particles such as gold, or so-called upconverting nanoparticles. Developed in its modern form by Dougherty in the 1970’s [17], PDT is currently clinically employed in dermatology (e.g., for actinic keratinosis), ophthalmology (e.g., for age-related macular degeneration) or oncology (e.g., for skin, retina, bladder, gastronintestinal, prostate, lung, head and neck cancers). However, as reported by Zhang et al. in a recent review [18], clinical development of PDT remains somewhat limited because of various challenges, ranging from photosensitizer formulation, light dosimetry, to planning and monitoring the treatment [15,18–22]. Some of these points have been recently reviewed: ideal photosensitizers [23], challenges in formulating photosensitizers, and choosing the right light dosimetry [24], as well as monitoring the treatment response [25]. Among all these, the adaptation of light source and dosimetry is currently a very active field. Indeed, protocols adapting the irradiation are tested based on daylight or continuous [26] low irradiation, or using special devices such as fabrics [27] or catheters. The light sources are also diversified from lasers (range of 1–7 W) to diode lasers (2–2.5 W) or LEDs enabling the use of much lower energies. Another point raising much interest in nanomedicine is linked to an optimized biological model that enables to limit in vivo experiments in accordance with the “3 R’s” rule of animal testing ethics (the “3 R’s” stand for replacement, reduction and refinement aiming at limiting the number of in vivo experiments requiring the sacrifice of animals). 3D systems such as spheroids or cell-derived matrices and using microfluidics have thus been suggested for PDT [28–29].

Based on this existing literature, this review will first present the state-of-the-art (typically since 2014) of block copolymers used for PDT. However, our aim is also to provide an analysis of the methods already used or applicable to improve the efficiency of the nanocarriers. This will be the subject of the second part. A third part will focus on the interactions between the vectors with the cell membrane, either in its native form or in biomimetic models. Finally, cellular entrance processes and intracellular targeting will also be described, showing possible intracellular targeting methods as well as the use of irradiation to promote drug delivery (photochemical internalization).

### Block copolymers used for vectorization of photosensitizers

**Figure 3:**
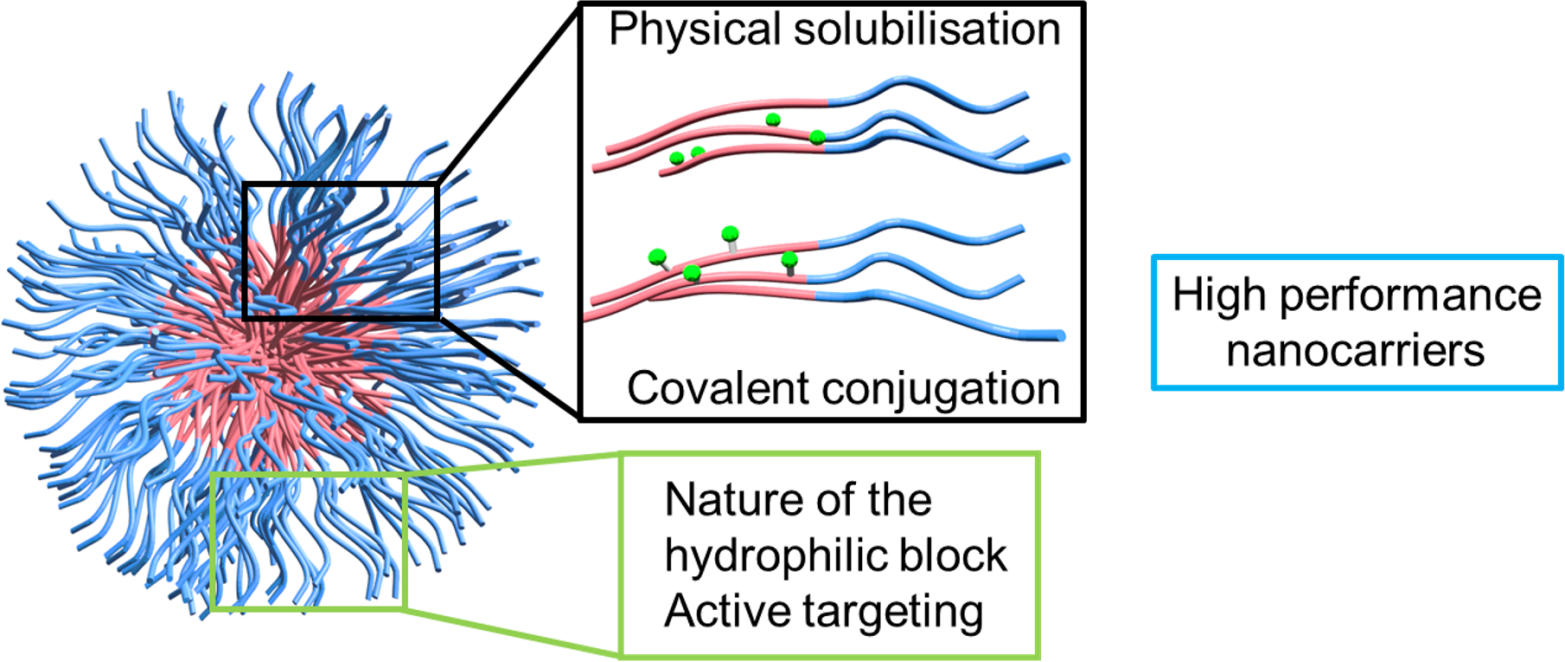
Schematic representation of the strategies used for delivery of photosensitizers using block copolymer self-assemblies.

Most of the used photosensitizers are highly hydrophobic and have the tendency to aggregate in aqueous environments, which is detrimental for their effectiveness in PDT. Block copolymer nanoassemblies offer the unique possibility to protect the photosensitizer in a hydrophobic environment (as described in Figure 3) and to prevent the aggregation. At the same time, they improve the biodistribution, pharmacokinetics and photochemical reactivity of the photosensitizer. Thus, typical PDT side effects, i.e., patient skin photosensitivity, can be avoided.

The hydrophilic block of the copolymers will influence the interactions with the surrounding biological media and, in particular, will play a role in the distribution in the body and in cells. The properties of the hydrophobic block can be tailored in order to guarantee a good insertion of the photosensitizer in the nanoobjects and confer the nanovectors with specific functionalities.

**Figure 4:**
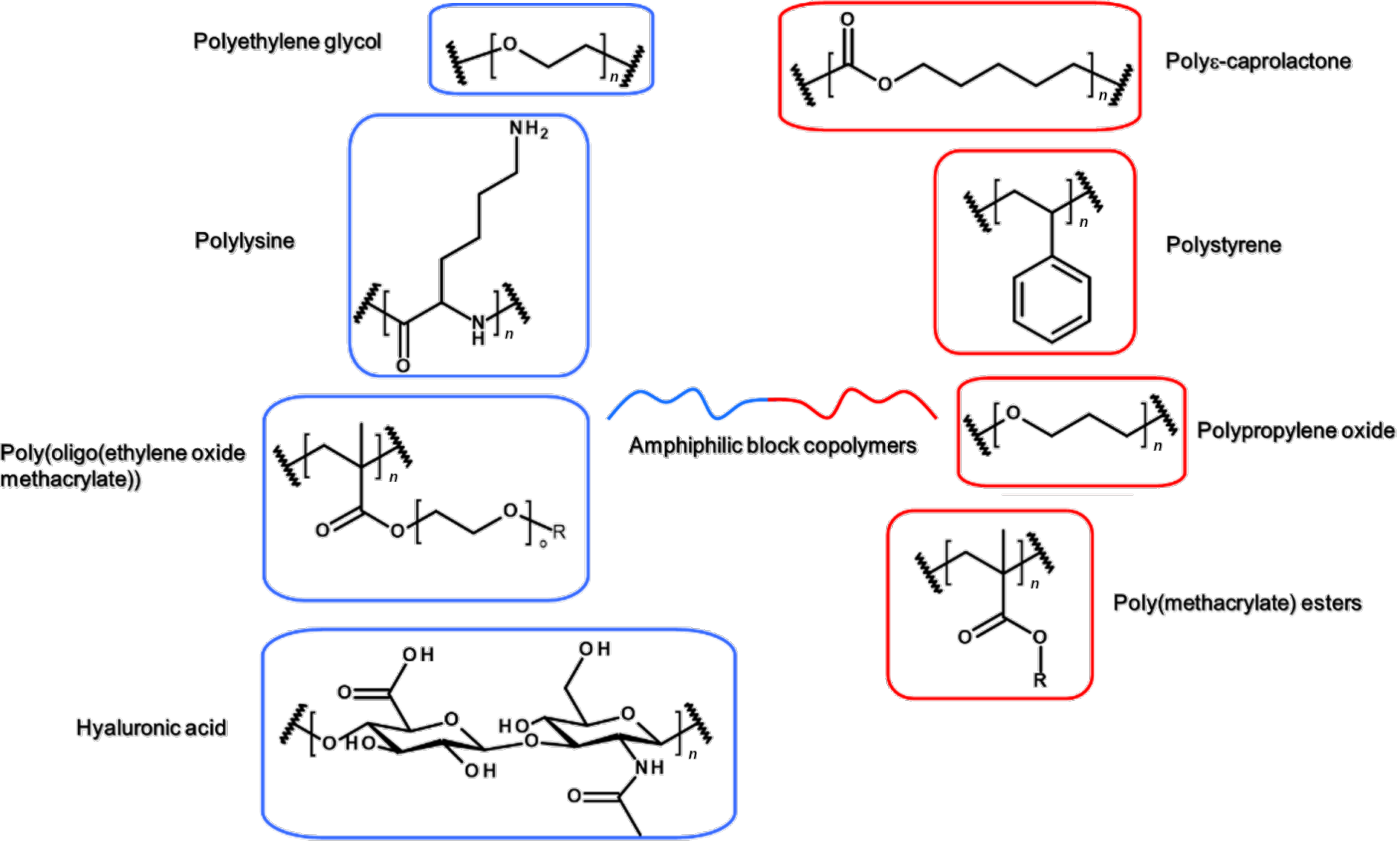
Chemical structures of the main blocks commonly described in recent literature.

The development of polymer engineering has allowed for the design of sophisticated structures that can be varied at will. In the following section we will discuss the block copolymer structures that have been proposed in the literature for PDT in the last years. The chemical structures of some key blocks used for self-assemblies are described in Figure 4. We will first present the structures for the hydrophobic block and then the ones for the hydrophilic block. In the former case, the photosensitizer can be simply solubilized in the interior of the self-assemblies or covalently linked to the copolymer backbone.

#### Physical solubilization of the photosensitizer

The structures, features and applications of block copolymers used for the physical solubilization of photosensitizers are described in this section and summarized below in Table 1. Most commonly applied are biocompatible, nontoxic and FDA-approved copolymers, such as pluronics [30–35] or biodegradable aliphatic polyesters, based for instance on ε-caprolactone or lactic acid [36–41]. In particular, the degradation of polyesters in vivo, a combination of both hydrolytic and enzymatic processes, makes them a first choice for the controlled delivery of drugs [42].

Polymers with an acrylate backbone have been also used. The lateral chains can be functionalized in order to introduce functional groups for increasing the affinity to the photosensitizers and the loading efficiency, leading to a 100-fold phototoxicity improvement (in the case of the nicotinate group) [43]. Although not biodegradable, the poly(meth)acrylate backbone is known to be biocompatible, as demonstrated by its long use in opthalmology [44]. Counterintuitively, as it will be detailed in the next section, the introduction of aromatic units is not always an advantage [45].

In order to improve the solubilization of the photosensitizer, other interactions different from hydrophobic interactions have been proposed. For example, electrostatic interactions can improve the photosensitizer loading. Mostly amino acid-based polymers with poly(ʟ-lysine) [46–47] or poly(aspartic acid) [48–49] charged blocks have been employed for poly ion complex assemblies (PICs). This strategy also revealed not to be always appropriate for ROS production and in the case of vinylpyridinium-based block copolymers (with both the pH-insensitive 4-vinilpyridinium and the positively charged *N*-methyl-2-vinylpyridinium iodide), the PIC micelles are even proposed as antioxidants due to the formation of H aggregates between photosensitizer molecules, which hampers the production of singlet oxygen [50–51]. Hence, a careful choice of the hydrophobic block of the copolymer–photosensitizer couple for electrostatic interactions is needed. Interestingly, host–guest complexation of porphyrins in the cavities of cyclodextrin was also proposed as driving force of amphiphilic self-assemblies [52–54].

A main drawback of this kind of nanosystems in which the photosensitizer is simply dissolved in the hydrophobic environment is the possible premature leakage during body circulation (enhanced by degradation) with a consequent lower drug concentration at the target site or other side effects. To tackle this problem, systems responsive to biological signals or containing a covalently linked photosensitizer have been proposed as a solution. The latter strategy will be described below in a dedicated section. In the following, we will focus on responsive photosensitizer-loaded nanosystems.

In such nanosystems, characteristic properties of the tumor microenvironment (endogenous trigger) or an external trigger can act as a stimulus and bring a structural modification of the block copolymer influencing the self-assembly behavior and, consequently, the photosensitizer loading ability. Typical endogenous triggers for pH- and redox-responsive drug delivery are i) acidic tumor tissues (pH 6.0–7.0), endosomes (pH 5.0–6.0) and lysosomes (pH 4.0–5.0) microenvironments, ii) high intracellular glutathione concentration (ca. 10 mM), and iii) very recently, tumor hypoxia (i.e., low oxygen concentrations due to rapid use of blood supply for cancer cell growth) [55]. A typical exogenous trigger is light that can induce the cleavage of covalently linked groups and the solubilization and degradation of the self-assemblies, followed by cargo release [56]. These well-known concepts in nanomedicine have been applied to photodynamic therapy applications.

A responsiveness to the pH value can be induced by inserting ionisable groups (such as amines and carboxylic acids for example) in the polymer backbone in order to induce a change in the nanostructure as a consequence of a change of pH value.

**Table 1:** Passive targeting and photosensitizers solubilized in the hydrophobic core. PIC: poly ion complex; PS: photosensitizer, FI: fluorescence imaging; PCI: photochemical internalization; PA: photoacoustic imaging; PTT: photothermal therapy.

block copolymer	specific feature	comments	ref

poly(ethylene oxide)-*block*-poly(propylene oxide)-*block*-poly(ethylene oxide)	–	in vitro (cancer lines, bacteria) and in vivo	[30,32–35,57]
poly(ethylene oxide)-*block*-poly[2-(methylacryloyl)ethylnicotinate]	–	osteosarcoma in vitro and in vivo	[43]
poly(styrene)-*block*-poly(acrylic acid)	–	adenocarcinoma in vitro	[58]
poly(butadiene)-*block*-poly(1-methyl-2-vinylpyridiniummethyl sulfate)-*block*-poly(methacrylic acid) and poly(ethylene glycol)-block-poly(ʟ-lysine)	PIC	lung carcinoma, in vitro and in vivo	[59]
poly(ethylene oxide)-*block*-poly(ʟ-lysine), poly(ʟ-lysine)-*block-*poly(ethylene oxide)-*block*-poly(ʟ-lysine)	PIC with PS	HUVEC and lung carcinoma, in vitro	[46–47]
poly(ethylene oxide)-*block*-poly(α,β-aspartic acid)/poly([5-aminopentyl]-α,β-aspartamide)	–	lung carcinoma, in vitro	[48]
poly(ethylene oxide)-*block*-poly(α,β-aspartic acid)	PIC with PS	lung carcinoma, in vitro	[49]
poly(*N*-methyl-2-vinylpyridinium iodide)-*block*-poly(ethylene oxide)	PIC with PS	–	[50–51]
branched polyethylene imine modified with perfluorooctanoic acid	O_2_ shuttle	cervix carcinoma, in vitro and in vivo	[60]
haemoglobin-conjugated poly(ethylene oxide)-*block*-poly(acrylic acid)-*block*-poly(styrene)	O_2_ shuttle	cervix carcinoma, in vitro	[61]
human serum albumin	O_2_ shuttle	adenocarcinoma and colon carcinoma, in vitro and in vivo	[62]
poly(oligo(ethylene oxide)methacrylate)-*block*-poly(β-benzyl-ʟ-aspartate)heptafluorobutylamine substituted	O_2_ shuttle	image-guided (FI), liver cancer, in vitro and in vivo	[63]
poly((ethylene oxide)methacrylate-*co*-poly(1*H*,1*H*,2*H*,2*H*-perfluorodecyl methacrylate))	O_2_ shuttle	lung carcinoma, in vitro	[64]
poly(oligo(ethylene oxide)methyl ether methacrylate)-*block*-poly(ʟ-lysine)	O_2_ production	image-guided (FI), liver and breast cancer, in vitro	[65]
methoxy-poly(ethylene oxide)-*block*-poly(ε-caprolactone)-benzyl	degradation	macrophages and endothelial cells, in vitro	[40]
poly(ethylene oxide)-*block*-poly(ε-caprolactone)	degradation	colon cancer and carcinoma, in vitro	[37,66–67]
poly(ethylene glycol)-*block*-poly(lactic acid)	degradation		[38]
poly(ethylene glycol)-*block*-poly(ᴅ,ʟ-lactide-*co*-benzyl glycidyl ether)	degradation	macrophage and kidney cells, in vitro	[45]
poly(ethylene glycol)-*block*-poly(ε-caprolactone)-*block*-poly[(2-(piperidin-1-yl)ethyl methacrylate]	O_2_ independent, pH-responsive	breast cancer, in vitro and in vivo	[36]
catalase/chitosan	O_2_ production, pH-responsive	carcinoma, in vitro and in vivo	[68]
BSA/poly(allylamine hydrochloride)	O_2_ production, pH-responsive	breast cancer, in vitro	[69]
poly(ethylene oxide)-*block*-[poly(4,5-dimethoxy-2-nitrobenzyl((5-methyl-2-oxo-1,3-dioxan-5-yl)methyl)carbamate)-*co*-poly(trimethylene carbonate)]	light-responsive	colon cancer, in vitro	[70–71]
poly(ethylene oxide)-*block*-poly(nitrobenzene-containing acetal)	pH- and light-responsive	PCI, cervix carcinoma, in vitro	[72]
arylboronic ester modified amphiphilic copolymer	ROS-responsive	chemo, breast cancer, in vitro and in vivo	[73]
poly(ethylene oxide)-*block*-poly(thioketal-containing 8,8-dimethyl-4,12-dioxo-3,13-dioxa-7,9-dithiapentadecane-1,15-diyldiacrylate)-*block*-poly(ethylene oxide)	O_2_ production, ROS-responsive	pancreatic cancer, in vitro and in vivo	[74]
methoxy PEG-Azo-poly(aspartic acid)-imidazole	responsive to ROS and hypoxia	lung cancer, in vitro and in vivo	[75]
methoxy poly[(ethylene oxide)-*co*-(aspartic acid)-imidazole]	ROS-responsive	breast cancer, in vitro and in vivo	[76]
adamantane-terminated 6-(5′-(4′-phenoxyl)-10′,15′,20′-triphenylporphyrin) and PEGylated cyclodextrin	redox responsive	breast cancer, in vitro	[77]
human serum albumin (intermolecular disulfide conjugation)	redox responsive	image-guided (FI, PA), PTT, kidney cells, breast cancer, in vitro and in vivo	[78]

Piperidine groups for example possess a p*K*_a_ value close to the acidity of tumor tissues and exhibit a transition from hydrophobic at pH 7.4 to hydrophilic at pH 6.8. As a consequence, micelles increase their diameter and ζ-potential values switch from negative to positive thus accelerating cellular internalization [36]. Poly ion complexes formed thanks to electrostatic interactions between positively charged weak bases and negatively charged weak acids are ideal pH-responsive nanocarriers. PICs formed by catalase and chitosan showed a stability change in response to the pH value. Between 7.4 in phosphate buffer (comparable to the cytoplasm environment) and pH 5.5 in acetate buffer (comparable to the lysosome environment) the diameter of nanoparticles decreased dramatically in the first 60 min [68].

**Figure 5:**
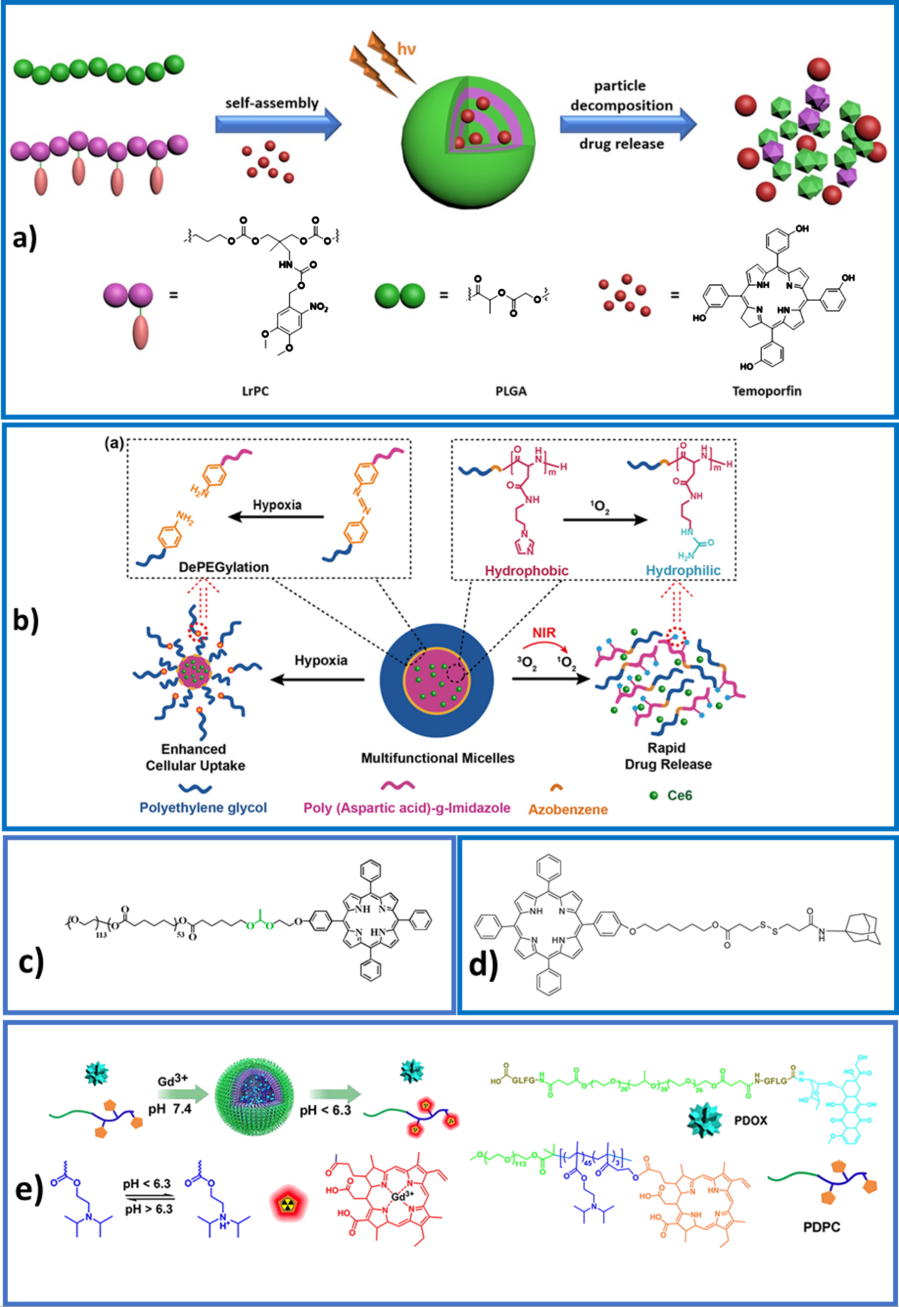
a) Light-responsive self-immolative polymers. Adapted with permission from [70], copyright 2018 American Chemical Society. b) Hypoxia- and ROS-sensitive polymers. Adapted with permission from [75], copyright 2018 American Chemical Society. c) An acetal-containing copolymer that can be hydrolyzed at low pH values. Adapted with permission from [79], copyright 2017 American Chemical Society. d) A disulfide link sensitive to redox conditions and to GSH concentration. Reproduced with permission from [77], copyright 2015 The Royal Society of Chemistry. e) A pH-sensitive polymer. Adapted with permission from [80], copyright 2016 American Chemical Society.

The reducing power of glutathione (GSH) was exploited in a supramolecular micellar system formed through the host–guest interaction between a PEGylated cyclodextrin and adamantane moieties conjugated to a porphyrin photosensitizer through a disulfide bond. Once the disulfide link was cleaved by glutathione, the porphyrin photosensitizer was released and the size of the nanoobjects in solution increased (Figure 5d) [77]. For a combined photodynamic therapy/photothermal therapy (PDT/PTT) approach, indocyanine green (ICG) has been encapsulated in a protein, namely human serum albumin. First human serum albumin (HSA) is reduced and encapsulates ICG thanks to electrostatic interactions, then the disulfide links are reconstructed for carrier stabilization. This carrier is then glutathione-sensitive and its reduction under in vivo conditions enhanced the PDT efficiency [78].

The cleavage of ROS-responsive groups, such as arylboronic ester lateral groups [73] or thioketal moieties connecting the hydrophilic and hydrophobic blocks [74], were used to alter the hydrophilic/hydrophobic balance and cause the disassembly of the nanoobjects. In another work, amphiphilic block copolymers were obtained by introducing an imidazole functionality on a poly[(ethylene glycol)-*co*-(aspartic acid)] backbone. The imidazole group can act as a singlet oxygen scavenger, which will transform it into a urea moiety. Upon light excitation, the encapsulated chlorin-e6 produces ROS species and the urea formation induces a size expansion of the self-assemblies and a rapid release of the photosensitizer (Figure 5b) [76].

In an original approach, light-responsive self-immolative polymers [70–71] (Figure 5a) based on a polycarbonate backbone have been proposed. Here a photolabile *o*-nitrobenzyl group can be removed through a redox photoisomerization process, leading to the release of a functional amine group inducing intramolecular cyclization. The biological safety of the degradation products has been evaluated.

A quite new concept consists in exploiting hypoxia exacerbated by oxygen consumption during PDT to make nanoobjects work. By combining both hypoxia- and ROS-sensitive groups, a methoxy poly(ethylene oxide)-*block*-poly(aspartic acid) copolymer functionalized with imidazole side chains formed so-called multi-compound micelles. Their internalization could be improved thanks to the deshielding of poly(ethylene oxide) induced by hypoxia which acted on the azobenzene link between the hydrophobic and hydrophilic blocks. Imidazole groups are ROS-sensitive and can be oxidized into urea thus guaranteeing photosensitizer release in intracellular environment (Figure 5b) [75].

#### Covalent conjugation of photosensitizers

As mentioned above, the physical solubilization of the photosensitizer suffers from the risks of leakage from the nanocarrier before the target is reached. Leakage can be prevented when the photosensitizers are covalently linked to the polymer backbone. However, the photosensitizers are mostly inactive in the self-assemblies when covalently linked and a precise stimulus is needed at the target site in order to activate the photosensitizers and produce ROS under illumination. Recent developments are summarized below in Table 2.

Different strategies have been applied in order to produce singlet oxygen on demand [81]. Among them self-quenching and Förster resonance energy transfer (FRET) quenching are the most frequently used in self-assembled nanocarriers. An alternative possibility to avoid quenching is to mix the photosensitizer-conjugated polymer with a polymer without photosensitizers [82]. Interestingly, the effect of the position of the photosensitizer in the polymer backbone is not trivial and it is an element one can play with in order to maximize the phototoxicity with minimal amounts of photosensitizer as will be discussed in the next section [82].

Self-quenching, also known as aggregation-induced quenching, is typical in nanosystems where the presence of the photosensitizer is the main driving force of the self-assembly. The photosensitizer is then concentrated in the hydrophobic core in a dormant state, due to the formation of π−π-stacked aggregates, that is not phototoxic under illumination. In order to make these systems work, the monomeric state of the photosensitizer has to be restored by a trigger. The first examples of this approach are enzymatically activated copolymers, mostly based on polylysine [83–84]. In these examples chlorin-e6 could be conjugated to the backbone thanks to reactive amine bonds. Not all of the lysine units should be modified to guarantee water solubility and enzymatic activation [83]. PEGylation could also improve solubility, but it was also proved to be detrimental regarding quenching [84]. More recently, polysaccharides based on chitosan or heparin have also been considered [85–86].

**Table 2:** Passive targeting and photosensitizers covalently linked to the hydrophobic block. PET: positron emission tomography; FI: fluorescence imaging; MRI: magnetic resonance imaging; NIRFI: near-infrared fluorescence imaging; PTT: photothermal therapy; FRET: Förster resonance energy transfer; PA: photoacoustic imaging; ROS: reactive oxygen species; AIE: aggregation-induced emission; PIC: poly ion complex; PS: photosensitizer.

polymer	specific feature	comments	ref

hybrid telodendrimers comprising linear polyethylene glycol and dendritic oligomers of pyropheophorbide a and cholic acid	redox (self-quenched)	chemo, PET, image-guided (FI), MRI, ovarian and lung cancers, in vitro and in vivo	[87]
poly(ethylene glycol)-*block*-poly(disulfide ester 5-(4-(6-hydroxyhexyl)phenyl)-10,15,20-triphenylporphyrin)-*block*-poly(ethylene glycol)	redox (self-quenched)	lung cancer, in vitro	[88]
biarmed poly(ethylene oxide)-(pheophorbide a)_2_	redox (self-quenched)	adenocarcinoma, in vitro	[89]
doxorubicin and Zn phthalocyanine conjugated to methoxy polyethylene glycol-*block*-poly(β-benzyl-ʟ-aspartate)	pH value and redox (self-quenched)	chemo, liver cancer, in vitro and in vivo	[90]
poly(ethylene glycol)-*block*-poly(ε-caprolactone)-*alt*-porphyrin	pH value (self-quenched)	chemo, lung cancer, in vitro	[79]
poly(ethylene glycol)-*block*-poly(γ-benzyl-ʟ-glutamate)	(self-quenched)	adenocarcinoma and melanoma, in vitro	[82]
Ce6-poly(ethylene glycol)-*block*(azo)-poly(ε-caprolactone)	hypoxia (self-quenched)	chemo, adenocarcinoma, in vitro	[91]
poly(hydroxypropyl methacrylamide) conjugated pyropheophorbide a	(self-quenched)	NIRFI, colon cancer and melanoma, in vitro and in vivo	[92]
Ce6-conjugated poly(ethylene glycol)-*block*-poly[(diisopropylamino ethyl methacrylate-*co*-hydroxyl methacrylate)]	pH value	chemo, PTT, MRI, PA, NIRFI, breast cancer, in vitro and in vivo	[80]
camptothecin and protoporphyrin IX conjugated to dextran	pH value, redox	chemo, pancreatic cancer and endothelial cells, in vitro and in vivo	[93]
poly(*N*-isopropylacrylamide)-*block*-poly(6-(5′-(4′-phenoxyl)-10′,15′,20′-triphenylporphyrin) methacrylate)	temperature	breast cancer, in vitro	[94]
hyperbranched conjugated polymer core and thermoresponsive hyperbranched polyether shell	FRET, temperature	PTT, adenocarcinoma, in vitro and in vivo	[95]
tetraphenylethenethiophene-thioketal-poly(ethylene oxide)	ROS	image-guided (AIE), chemo, breast cancer, in vitro	[96]
Zn porphyrin conjugated to poly(oligo(ethylene oxide)methyl ether methacrylate)-*co*-poly(trifluoroethyl methacrylate)	antihypoxia	carcinoma and melanoma, in vitro	[97]
salicylaldazine hexadecane-*block*-poly(ethylene oxide)	–	image-guided (AIE), adenocarcinoma, in vitro	[98]
modified poly(oligoethylene oxide)-alt-octadecene	–	image-guided (FI), PET, breast cancer and glioblastoma, in vitro and in vivo	[99]
poly(triphenylphosphonium-(2-hydroxypropyl)methacrylamide)-*co*-poly(*N*-(2-hydroxypropyl)methacrylamide)-*co*-poly((2*Z*,2'*Z*)-3,3'-(2,5-bis((4-methylacrylate)(phenyl)amino)-1,4-phenylene)bis(2-(3,5-bis(trifluoromethyl)phenyl)acrylonitrile))	–	image-guided (AIE), lung and neck cancer, in vitro	[100]
poly[(poly(ethylene glycol)methyl ether methacrylate)-*co*-(3-aminopropyl methacrylate)]-*block*-poly(methyl methacrylate)	–	image-guided (FI, PA), MRI, breast cancer, in vitro and in vivo	[101]
poly(styrene‐*co*-5,10,15,20‐tetrakis(pentafluorophenyl)porphyrin)‐*block*‐poly(ethylene oxide monomethyl ether acrylate)	–	glioblastoma, in vitro	[102]
poly(ethylenimine)-beta-carotene conjugate and pheophorbide a modified heparine (PIC)	scavenger “quenched”	breast cancer, in vitro	[103]
porphyrin conjugated poly(ethylene oxide)-*block*-poly(pentafluorophenyl methacrylate)	antihypoxia	liver cancer, in vitro	[104]
catalase-meso-tetra(*p*-hydroxyphenyl)-poly(ethylene oxide)	O_2_ production	breast cancer, in vitro and in vivo	[105]
poly[oligo(ethylene oxide) methyl ether methacrylate]-*block*-poly(ortho-substituted 9,10-diphenylanthracene methacrylate-*co*-*n*-hexyl methacrylate tetraphenyl porphyrin-*co*-*n*-butyl methacrylate)	singlet oxygen production without PS	PTT, liver cancer, in vitro and in vivo	[106]

Another stimulus for activation used in self-quenched self-assemblies is the reduction of the disufide bond by gluthathione. This approach has been used to chemically link the photosensitizer molecule pheophorbide a via a disulfide bond to the two arms of a methoxy poly(ethylene oxide) [89], or to the aspartate backbone of a poly(ethylene oxide)-*block*-poly(β-benzyl-ʟ-aspartate) [90]. The latter example is proposed for chemotherapy as doxorubicin is also chemically linked through an acid-labile hydrazone linker to the aspartate backbone. In another recent example the hydrophobic central block was made of porphyrin molecules linked by disulfide groups; in the intracellular microenvironment the reduction by glutathione could activate the porphyrin molecules for PDT [88].

For self-assembled nanoparticles that are too labile and easily disassemble in vivo, cross-linking with disulfide bonds has been proposed for stabilization. Li et al. [87] proposed telodendrimers formed by linear polyethylene oxide and pheophorbide a and cholic acid at the ends of dendritic polylysine. The insertion of four cysteine mioeties in the oligolysine backbone allowed for a stabilization of the nanoparticles through disulfide bonds and conferred a sensitiveness to GSH at intracellular level. The authors showed that the intact micelles generated heat upon irradiation, thus allowing PTT, while fluorescence and ROS generation were the main deactivation processes in the case of disassembled micelles. This is an example of “all in one“ nanomedicine used for chemotherapy combining loading with doxorubicin, PDT and PTT. This is possible thanks to the activation of the photosensitizer and multimodal imaging using the fluorescence of the photosensitizer (near-infrared fluorescence imaging, NIRFI) and the addition of Gd^3+^ ions (magnetic resonance imaging, MRI) or ^64^Cu^2+^ (positron emission tomography, PET).

A pH-sensitive acetal bond between poly(ε-caprolactone) and porphyrin was used to release porphyrin at pH 5 (Figure 5c) [79]. In a poly(ethylene glycol)-*block*-poly(ε-caprolactone) polymer conjugated with chlorin-e6, an azobenzene group that can be cleaved at very low oxygen concentrations links the hydrophobic and the hydrophilic block. Upon irradiation and depletion of oxygen due to the PDT activity of chlorin-e6, the block copolymer nanovector disassembled and the anticancer drug, doxorubicin, was released [91].

The photoactivity of the photosensitizer can also be modulated by conjugation with a quencher molecule different from the photosensitizer itself. IR780 could be used as a quencher of chlorin-e6 fluorescence in albumin-based nanosystems [107]. Upon NIR excitation and IR780 degradation chlorin-e6 is activated.

In an original way, Huang et al. exploited FRET activation in a reverse manner [95]. The photosensitizer is covalently linked to a thermo-responsive hyperbranched polyether shell, which keeps it far away from the hyperbranched conjugated core. Upon NIR excitation of the core block, because of the photothermal effect, the shell shrinks thus bringing the photosensitizer closer to the core allowing for fluorescence resonance energy transfer and singlet oxygen production. In other proposed polymers, fluorescence quenching is not discussed, but in vitro studies prove the higher phototoxicity of the covalently linked photosensitizer [94,102].

An original way of preventing ROS production before reaching the target site is the use of a scavenger. In the work from Li et al. [103], a PIC is formed between a negatively charged heparine modified with pheophorbide a and a positively charged polyethyleneimine coupled to β-carotene. After disassembly of the nanoparticles, the mean distance between pheophorbide a and β-carotene increases thus activating PDT.

In an alternative strategy, aggregation-induced emission (AIE) fluorophores have been proposed as a solution to aggregation-induced quenching. These luminogens are characterized by high emission and efficient ROS production in the aggregated state under light irradiation, which is why they can be used for image-guided PDT [96,98,100]. As an example, tetraphenylethenethiophene (TPETP) conjugated to PEG through an ROS-sensitive thioketal link was proposed to overcome the drug resistance of cancer cells. Indeed, it induced membrane permeability of the endo-lysosome and particle disassembly after white-light irradiation thus triggering the release of doxorubicin in the cytosol [96]. In the study by Zheng et al. [100] the AIE fluorophore is used as cross-linker and increases the aggregates stability.

#### The hydrophilic blocks

**Chemical compositions.** For many years, polyethylene oxide (PEO) also referred to as poly(ethylene glycol) (PEG) has been the favorite hydrophilic component in copolymers whenever a biological application was sought. Indeed, one of the key features of PEO is to provide (steric) stabilization by excluding other macromolecules and particles due to the high flexibility and large exclusion volume of PEO strands in water. This imparts biocompatibility and prolonged circulation time to the objects by minimizing the adsorption of proteins and adhesion to cells [108–109]. A hydrophobic cargo, well within the hydrophobic core is thus protected from hydrolysis and enzymatic degradation. Besides, PEO prevents the recognition from the mononuclear phagocyte system and preliminary clearance from the bloodstream is reduced. Although PEO has been widely available to chemists around the world, its actual synthesis remains a task for specialists. Most often, as in the work described by Ibrahimova et al. [82], PEG is assembled as a pre-synthesized block. Astute chemists have managed to assemble complex architectures, such as the multicompartment nanovectors described by Synatschke et al. [59], where the combination of polyionic complexes and amphiphilic polymers lead to bottlebrush-on-sphere assemblies.

Numerous examples can be found for nanovectors for PDT sensitizers having a PEO hydrophilic block. Pluronics, for example, are ABA triblock copolymers where block A is PEO and block B is poly(propylene oxide) PPO. Pluronics-based structures have been explored extensively in PDT applications and continue to garner attention, as in the study by Py-Daniel and co-workers [57].

In a recent study, Vilsinki et al. [58] used polyacrylate as a hydrophilic block, effectively rendering the self-assemblies highly negatively charged at physiological pH values. Indeed, negatively charged nanoparticles are known to be capable of evading the mononuclear phagocyte system and enjoy prolonged blood circulation [58,110].

**Active targeting through hydrophilic block.** Recent efforts in targeting through the hydrophilic block are summarized in Table 3. Carbohydrates have been used in order to confer targeting properties and they are often modified by grafting the hydrophobic photosensitizers in order to yield the amphiphilic properties necessary for self-assembly [93,111–112]. Among the targeting molecules, hyaluronic acid [111–112] is known to interact with CD44 over-expressed by some tumor cells. Mannitol [113] or galactose [114] have been used for their inherent biocompatibility and bioadhesive/targeting properties. Interestingly mannitol derivatives could be obtained with an environmentally friendly strategy using a lipase for sugar transesterification.

**Table 3:** Polymers used for active targeting. FI: fluorescence imaging; PA: photoacoustic imaging.

polymer	specific feature	comments	ref

poly(ethylene glycol)-*block*-poly(lactic acid)-folate	–	ovarian cancer, in vitro and in vivo	[39]
hyaluronic acid-*block*-poly(ᴅ,ʟ-lactide-*co*-glycolide)	–	lung cancer, in vitro	[111]
chlorin-e6 conjugated hyaluronic acid	image-guided (FI, PA), stimulus by oxygen shuttle, redox (self-quenched)	breast cancer, in vitro and in vivo	[112]
poly(ᴅ-galactose methyl methacrylate)-*block*-poly[oligo(ethylene glycol) methyl ether methacrylate]-*block*-poly(carbobenzoxy-ʟ-lysine)	image-guided (FI)	liver cancer and carcinoma, in vitro	[114]
poly(2,5-anhydro-3,4-di-*O*-benzyl-ᴅ-mannitol-*block*-poly(ethylene oxide); poly(2,5-anhydro-3,4-di-*O*-decanoyl-ᴅ-mannitol-*block*-poly(ethylene oxide); poly(2,5-anhydro-3,4-di-*O*-myristoyl-ᴅ-mannitol-*block*-poly(ethylene oxide)	–	lung cancer, in vitro	[113]
disulfide-containing poly(ε-caprolactone)-*block*-poly(ethylene oxide) mixed with biotinylated poly(ethylene oxide)-cypate	redox-responsive	liver cancer, in vitro and in vivo	[115]

Liu et al. have described a tri-block polymer system functionalized with galactose (PMAGP-POEGMA-Plys-Bodipy) [114]. Poly(oligo(ethylene glycol) methyl ether methacrylate) (POEGMA) plays the role of a hydrophilic shell for micelle stabilization, and the derivatized polylysin (Plys) acts as a hydrophobic core to load the photosensitizer (BODIPY), while PMAGP mainly serves to direct the target delivery to hepatoma cancer cells.

Folate (FA) has been extensively studied as a targeting moiety [116] due to the overexpression of folate receptors in a number of tumor types including ovarian [117] or breast cancers [118]. One of the most recent examples of FA use in PDT applications is by Li et al., where the authors have designed a FA-PEO-PLA construct to deliver hypocrellin B, a sensitizer extracted from fungi, to intraperitoneal tumors [39]. It was shown that the sensitizer concentration reached a maximum after 2 h in the targeted organs, as opposed to after at least 6–12 hours in other peritoneal organs, thereby creating a large window of opportunity for treatment with reduced side effects.

When targeting cell surface receptors, two strategies can be distinguished using antibodies directed against a chosen receptor, or using the ligand of the receptor itself. The group of Torchilin pioneered the use of antibody-based active targeting by copolymer self-assemblies [119], and applied it to the delivery of PDT sensitizers [120]. Many groups have explored this strategy since. More recently, one of the most often targeted cell-surface entities has been EGFR (epidermal growth factor receptor, overexpressed in a variety of solid tumors such as non-small cell lung cancer, head and neck carcinoma, ovarian, kidney, and pancreatic cancer). Chang et al. have explored the potential of PLA-PEG-AntiEGFR self-assemblies loaded with chlorin-e6 as a photosensitizer and found that this construct led to the increased internalization of the micelles through receptor-mediated endocytosis, which in turn led to increased cytotoxicity upon light activation [121]. Very recently, Zhang et al. have exploited the highly selective interaction between avidin and biotin to specifically target cells over-expressing the biotin receptor [115].

#### High-performance nanoassemblies

The current trends for polymer vector design point to the development of versatile and “all in one” nanocarriers embedding different functions in order to both visualize the tumor and kill it. For this purpose, PDT has been associated with multimodal imaging and other treatments such as chemotherapy or photothermal therapy (PTT). Doxorubicin or camptotecin, the most frequently used chemotherapeutic molecules, can be encapsulated [73,87,96,115] or chemically linked [80,90,93] to the polymer backbone and released under a precise stimulus. Chlorin-e6 [80], ICG [78], IR825 [80], IR 780 [106] or cypate [36,115] or pheophorbide [87] have been employed for the photothermal effect.

Regarding imaging, the tendency is to couple different techniques such as fluorescence, photoacoustic and magnetic resonant imaging. The photosensitizer itself can act both as a fluorescent probe and as a photoacoustic agent, for example chlorine-e6 [80,99,112] covalently bonded to the polymer backbone. Otherwise, IR825 [101] has been used in the interior of the polymer micelles for photoacoustic (PA) imaging. Other photosensitizers proposed for image-guided PDT are ICG [78], TCPP [101] and pheophorbide a-conjugated poly(*N*-(2-hydroxypropyl)methacrylamide) when irradiated at 680 nm [92]. Besides, the chelating properties of porphyrins towards ions such as Mn^2+^ [101] or Gd^3+^ [80] or ^64^Cu [99] can be used for magnetic resonant imaging (MRI).

**Oxygen self-compensation.** Local tumor hypoxia is one of the issues of PDT as the inefficient oxygen supply hampers the therapy efficiency based on the energy transfer to surrounding oxygen [122]. Moreover, oxygen consumption during the treatment exacerbates hypoxia conditions provoking PDT hypoxia resistance due to increase in tumor invasiveness and metastasis [123].

**Figure 6:**
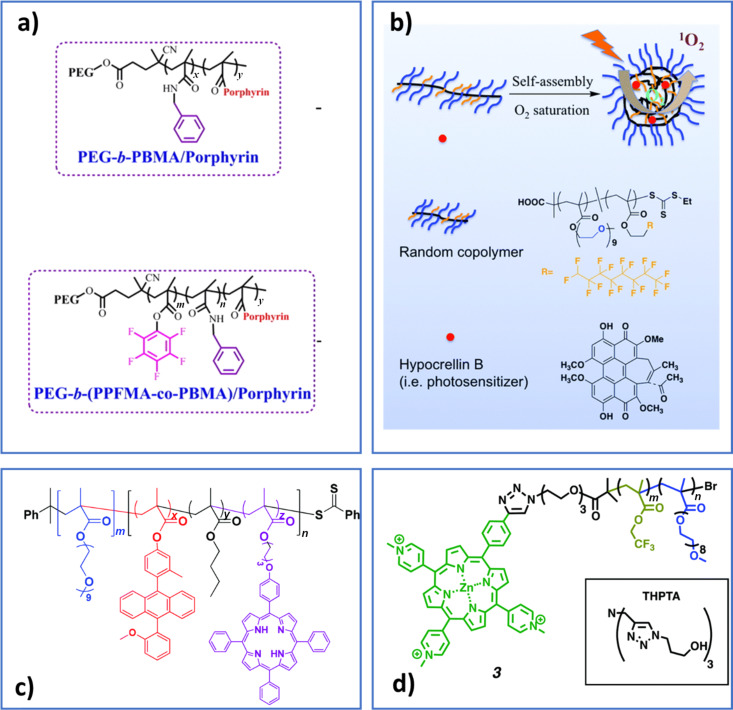
Block copolymers used as nanocarriers for overcoming hypoxia; a) adapted with permission from [104], copyright 2016 American Chemical Society, b) reproduced with permission from [64], copyright 2019 The Royal Society of Chemistry, c) reproduced with permission from [106], copyright 2018 The Royal Society of Chemistry, and d) reproduced with permission from [97], copyright 2017 The Royal Society of Chemistry.

In terms of the design of nanocarriers, different solutions have been recently proposed (see below in Figure 6) in order to (i) deliver oxygen in tumor tissues by using oxygen “shuttles”; (ii) produce oxygen in situ using chemical or photothermal reactions or (iii) circumvent the use of oxygen. Details of these solutions are as follows:

1. Taking inspiration from red blood cells, which transport oxygen via haemoglobin, the poly(acrylic acid) block in poly(ethylene glycol)-*block*-poly(acrylic acid)-*block*-polystyrene self-assemblies was conjugated to haemoglobin via carbodiimide chemistry [61]. The resultant carrier loaded with zinc phthalocyanine (ZnPc) was able to generate more singlet oxygen than the one without haemoglobin. Perfluorocarbon (PFC) is recognized for its biocompatibility, its ability to dissolve significant amounts of oxygen and to increase singlet oxygen lifetime [124]. A proposed solution is to stabilize a perfluorinated solvent by an hydrophilic shell, using lipids [125], albumin [62] or hyaluronic acid conjugated with chlorin-e6 conjugated [112]. In this way oxygen molecules can be absorbed in the core and the photosensitizer IR780 in the shell. A main drawback of this approach is extravasation from the nanoobjects, which can be avoided with perfluorinated block copolymers, which can simultaneously guarantee a high local concentration of PFC. Perfluorinated block copolymers are poorly soluble in water, thus a low total amount of fluorine was measured in the first reported examples (polymer concentration 0.1 mg·mL^−1^ in [104]). Efforts have been made in order to increase the solubility of fluorinated polymers by using charged poly(ethylene imine) stars (around 2 mg·mL^−1^ in [60]), for example. Another approach is to use random copolymers allowing for higher polymer concentrations (3 mg·mL^−1^ and up to 10 mg·mL^−1^ in [97] and [64] (Figure 6b), respectively). In [97] (Figure 6d) and [104] (Figure 6a) the photosensitizer was covalently linked to the polymer backbone; in both cases face-to-face H-type aggregation took place to some extent, nevertheless the higher oxygen concentration compensated it and singlet oxygen production efficiency was improved.
2. Polymer self-assemblies containing catalase [68] or MnO_2_ nanoparticles [65,69] have been developed as they can catalytically decompose endogenous H_2_O_2_ present in the tumor environment thus increasing the oxygen level in cancer cells. The electrostatic interactions between negatively charged catalase or bovine serum albumin and positively charged chitosan or poly(allylamine)-coated MnO_2_ have been exploited to obtain pH-sensitive nanovectors [68–69]. The low concentration of endogenous H_2_O_2_ together with the instability of catalase in physiological environments containing proteases, as well as the potential toxicity of Mn can be a limit. A nanocapsule where the catalase is protected by a brush-like PEO protective shell covalently linked to the photosensitizer meso-tetra(phydroxyphenyl) used as cross-linker was reported [105].
  In another elegant approach, polymer nanovesicles have been recently proposed as H_2_O_2_ reservoir [74]. In the aqueous pool of the nanovesicles, poly(amidoamine) (PAMAM) dendrimers conjugating chlorin-e6 and cypate were loaded together with H_2_O_2_. Upon NIR irradiation the cypate increased the temperature inducing the decomposition of H_2_O_2_ into oxygen.
3. An emerging strategy is to design nanocarriers able to transport reactive oxygen species in an inert form that can be activated once the vector reaches the tumors. Endoperoxides can be selected as a chemical source of singlet oxygen produced via thermal cycloreversion in an oxygen-independent manner [36,106]. This was possible thanks to the co-encapsulated cypate [36] or IR780 [106], which could induce hyperthermia through NIR irradiation. In [36], a 9,10-diphenylanthracene derivative is loaded while in [106] it is covalently linked to the methacrylate backbone together with the photosensitizer.

It might seem surprising that there is no special paragraph in this review to fully describe the observed PDT efficiency both in vitro and in vivo for each system presented here. Generally speaking, all polymeric vectors described in this review led to an improvement of the PDT efficiency. However, an overall comparison is meaningless, because for each research group the experimental parameters might be very different. To already many parameters in nanomedicine (including vector, cell line, 2D vs 3D, and in vitro vs in vivo conditions), the PDT conditions need to be added: irradiation source, wavelength of irradiation, irradiation power, total irradiance of the biological tissue. We therefore decide to let the reader examine each result depending on his standpoint and preferred to focus on the vector development philosophy. Nevertheless, the biological tests performed and the type of cancer examined are reported in Tables 1, 2 and 3 for the examples described in this section.

### Formulation optimization

As mentioned in the introduction of this review, there are many requirements an optimized nanovector needs to fulfil, i.e., biocompatibility, a controlled size between 20 and 200 nm, the highest possible loading, no release of the photosensitizer before the delivery site and an efficient ROS formation upon irradiation. To achieve this, the inherent properties of the photosensitizer itself are essential, but this is beyond the scope of this review, and the literature is rich on this point [23,37,126–130]. Our aim in this part is to focus on the vector and examine the different methodologies that can be used to optimize the final PDT efficiency. When using noncovalent encapsulation, the essential point is the relative interaction between the PS and its vector, compared to all competitive interactions in the biological medium. The proteins, lipids, extracellular matrix components are all ingredients that can transform or dissociate the vector, leading to the release of the PS. Increasing the stability of the vector can therefore be important, but some commercialized systems such as Abraxane^®^ use endogenous proteins such as human serum albumin (HSA) to take care of the drug traffic. In the case of PS and PDT, a first example has been described after a structural optimization of the PS (modified indocyanine) [131]. It is also noteworthy that human serum albumin has also been used in another study to deliver ICG, but a covalent disulfide bond was used to link HSA to the PS [78]. If a stable vector will have more chance to deliver its cargo to the appropriate site, a poor affinity between PS and the vector would be detrimental to the application, since this would lead to early release of the PS. Therefore, optimizing the PS/vector affinity is also of importance. But here again, a too stable vector might be problematic if the ROS produced upon irradiation are unable to reach the cellular components. This limitation is also the central one for PS covalently linked to the vector, as already mentioned before, and the disassembly of the nanovector is necessary. This short introduction clearly shows the very high complexity of vector development for PDT. In the subsequent paragraphs, we will thus examine each strategy for optimizing the vector formulation.

**Figure 7:**
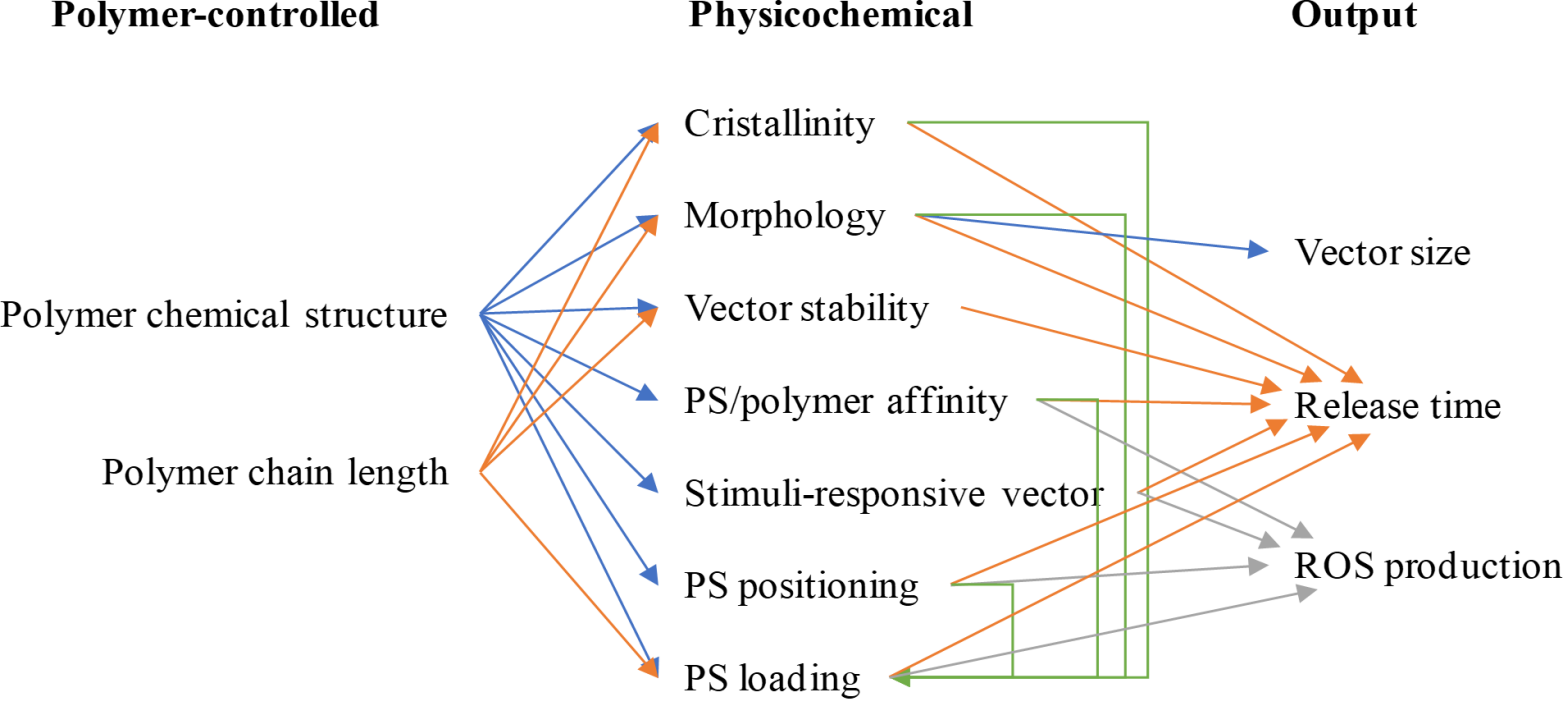
Schematic representation of the interplay between polymer structure, physicochemical characteristics, and their impact on PDT key parameters. PS: photosensitizer.

Figure 7 presents the different parameters that can be modified on the polymer itself, the links between them, and the specifications needed for the application.

The two central parameters that can be adjusted are the chemical structure of the polymer (including its chain morphology, either block, gradient or random and the comonomer ratios for copolymers) and the chain length, i.e., the molecular weight. The chemical structure will govern the prerequisite not mentioned in Figure 7, which is the biocompatibility. Obviously, a polymer that is not biocompatible will be rejected very early in the process of selection. Having said this, part 1 of this review showed the large variety of polymers used for PDT, going from aliphatic polyesters, polyacrylates to peptides or polysaccharides. The chemical structure will influence the crystallinity of the vector, its morphology (micelles, vesicles, or worm-like micelles), its stability ((bio)degradation or dissociation), the affinity to the PS and its loading and positioning inside the vector, and the possibility to introduce stimuli-responsive groups (enabling appropriate release). Similarly, the molecular weight of the polymer will have an impact on the crystallinity, the vector morphology, its stability and the PS loading. Most often, block copolymers are used but some points are noteworthy. For instance, Peng’s team has described the comparison between diblock and triblock copolymers based on polylysine (Plys) and PEO for PIC formation and they showed that Plys-PEO-Plys triblock was better than the diblock for PDT [46]. Regarding the ratios between the different comonomers, a thorough study assessed the encapsulation of chlorin-e6 in vectors based on pluronics exhibiting a large range of hydrophilic–lipophilic balance (HLB) and showed that an optimal HLB existed for a high PDT efficiency [35].

#### Cristallinity

Albertsson’s team published a study comparing semi-crystalline to amorphous vectors based on ε-caprolactone (CL), ʟ-lactide (LA) or ε-decalactone (DL) copolymers. All polymers formed micelles ranging from 25 to 60 nm but only those incorporating DL were amorphous. The study showed that the critical aggregation concentration was higher for amorphous systems and that the loading of aniline pentamer was better in the amorphous vector [132–133]. A similar loading improvement in amorphous vectors for indomethacin was described by Alexander and co-workers [134].

#### Morphology/size

Morphology includes both the assessment of the shape and the difference between micelles and vesicles, both being spherical but, respectively, hydrophobic or hydrophilic at their core. This is very rapidly linked to the vector size, since micelles will exhibit a typical size of 10–30 nm, vesicles will be typically larger than 60 nm. It is furthermore important to point out that the morphology of polymer self-assemblies is far from clear. Whereas the morphology of lipidic assembled systems is quite simple, the unambiguous determination of the morphology of polymeric systems is complicated. Small objects based on amphiphilic polymers with a size typically smaller than 30 nm can be described as micelles (hydrophobic core, hydrophilic corona) and this is confirmed by TEM and radiation scattering experiments (either light or X-rays or neutrons). For polymer vesicles, cryo-TEM images yield often doubtless morphology information. However, there are numerous systems that cannot be described as micelles or vesicles. Such cases are for instance large compound micelles constituted of small micelles [135–136]. The definite morphology assessment of the assembly needs the use of cryo-TEM or scattering techniques and is time-consuming. This explains why the literature is full of examples where authors might indicate micelles as a generic term or just nanoparticles without giving technical proof of the actual morphology.

**Figure 8:**
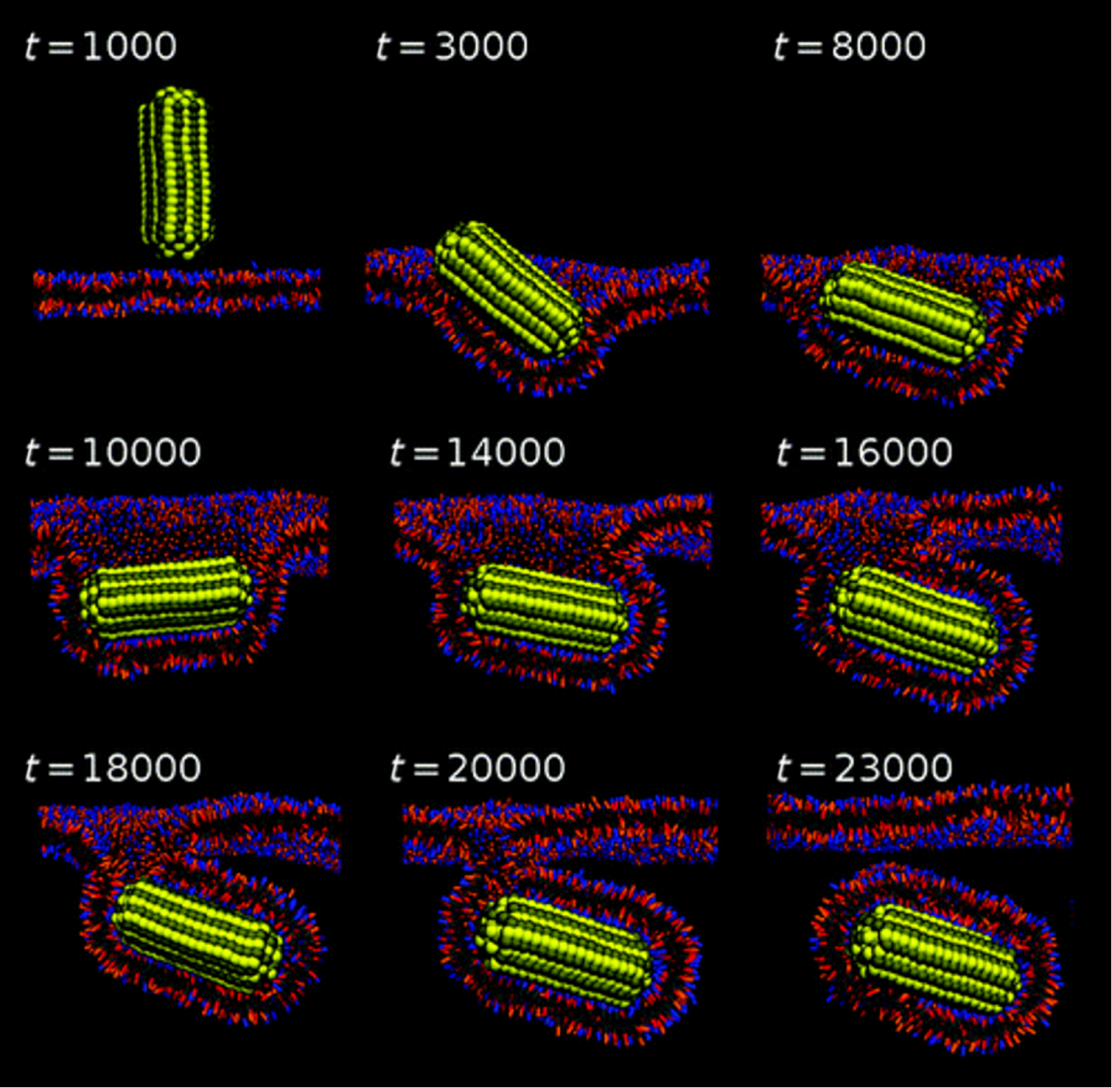
Representative snapshots describing the endocytosis pathway for spherocylindrical nanoparticles. Reprinted with permission from [141], copyright 2011 American Chemical Society.

Regarding the shape, a fundamental study was that of Discher on PEO-PCL vectors, which pointed at a higher efficiency using elongated vectors compared to spherical ones [137–139]. Simulations [139–141] showed that from a thermodynamic standpoint it is always more favorable to encapsulate spherocylindrical particles instead of spheres with the same radius and that endocytosis of spherocylinders occurs with parallel alignment to the membrane surface (Figure 8). Recent reviews have shown the importance of controlling the shape of the vectors, both for cell penetration but also for the behavior in the blood stream [142]. Regarding PDT more specifically, Till et al. examined the PDT efficiency of pheophorbide a when incorporated in micellar, vesicular or worm-like PEO-PCL vectors in two cell lines (FaDu or HCT 116) [66–67]. No strong improvement was observed for elongated systems working on 3D spheroids and, interestingly, a micelle/vesicle mixture led to synergistic effects on HCT 116 cells but antagonistic effects for FaDu cells [67].

#### Vector stability

The stability has to be assessed from two standpoints: the degradation of the polymer itself and the possible dissociation of the vector in the biological medium. Regarding degradation, the polymer should be stable enough for the application period, which is typically from a few hours to two days, corresponding to the usual time to benefit from the EPR effect. This stability specification is therefore not very demanding and most recent polymers fulfill it. In contrast, the possible dissociation upon confrontation to biological media is an essential point that has been examined from the very beginning of nanomedicine [11]. The major concern based in the case of lipidic vectors was that polymeric vectors might dissociate upon sudden dilution following injection. While this might be true for systems at thermodynamical equilibrium, it is not any more for kinetically frozen vectors, which is very often the case for polymeric self-assemblies. Thus, for polymeric self-assemblies, we cannot talk anymore of critical micelle concentration (cmc) but only of critical aggregation concentration (cac) as the threshold for which their formation is observed. A typical cac range for amphiphilic block copolymers used in nanomedicine is a few milligrams per liter, which represents roughly the micromolar range. This has to be compared to cmc values of small surfactants, which are typically closer to the millimolar range. Furthermore, an important point is that dissociation of the self-assembly will not necessarily occur as soon as the concentration drops below the cac, owing to a kinetic lag linked to the low mobility of the polymer chains, as demonstrated in an early study of Kataoka et al. who showed that dissociation took place over several days [143]. The most frequently suggested solution to avoid any dissociation upon dilution is cross-linking of the vector. For PDT and other therapeutic cases this has been also described [66], showing a strong improvement of the treatment. The PS can be used both as ROS generator and also as cross-linker [101].

**Figure 9:**
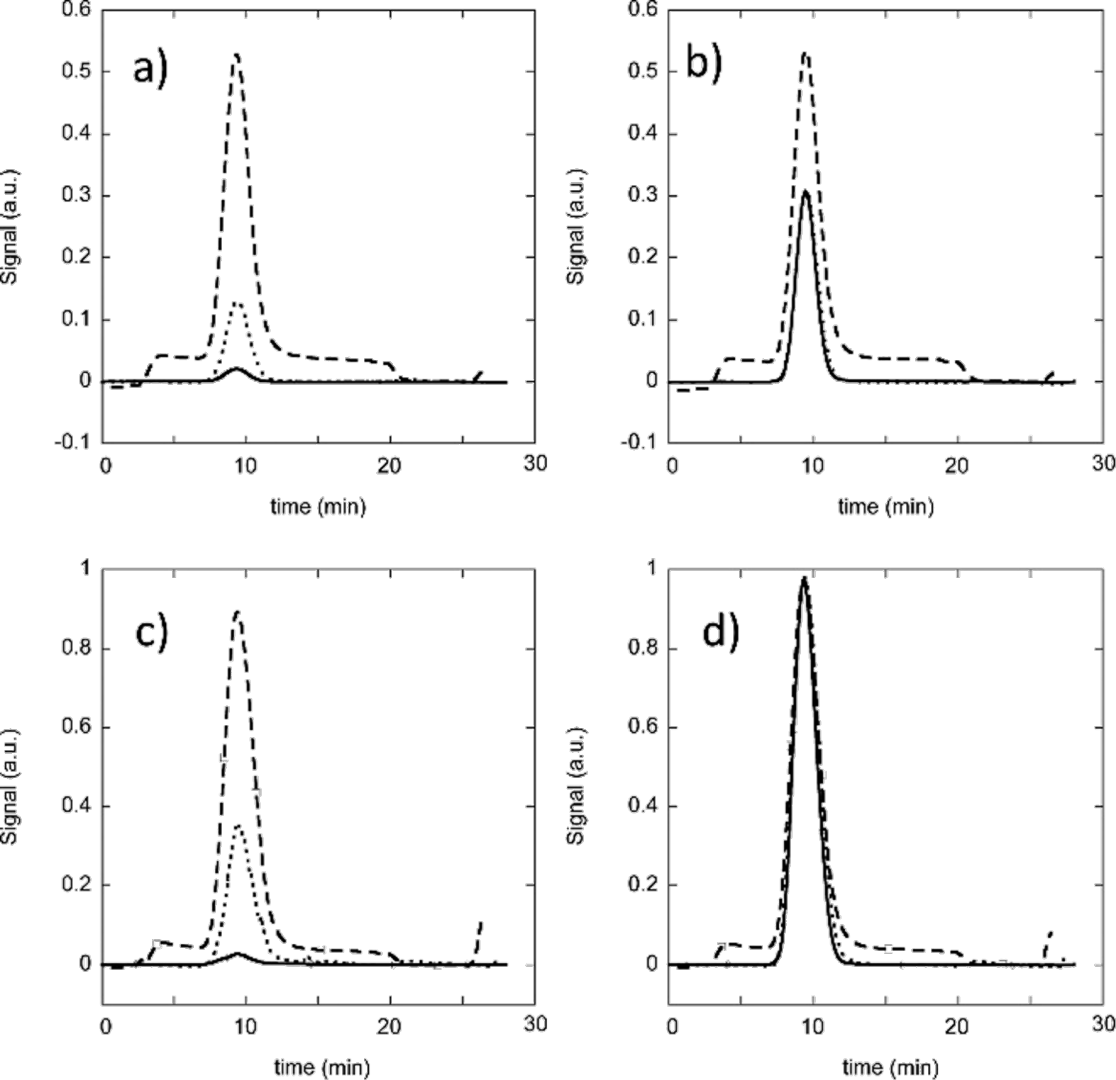
Field flow fractograms of PEO(2400)-*b*-PDLLA(2000) and PEO(3100)-*b*-PS(2300) micelles. The multi-angle light scattering (MALS) signal is represented by the dotted line, the RI signal by the dashed line, and the absorption at 412 nm by the full line. (a) Empty PEO(2400)-*b*-PDLLA(2000), (b) PEO(2400)-*b*-PDLLA(2000)/Pheo, (c) empty PEO(3100)-*b*-PS(2300), and (d) PEO(3100)-*b*-PS(2300)/Pheo. Reprinted with permission from [29], copyright 2014 American Chemical Society.

Regarding the stability in biological media, different conditions have been described, going from exposure to single proteins to the harshest one being fetal bovine serum (FBS). FRET follow-up has been often performed to examine the stability of polymeric self-assemblies and this review will only cite a few recent examples based on various techniques, which examined the stability of polymeric vectors using field flow fractionation, enabling therefore an efficient separation between the proteins of the medium and the vectors [45,144]. As exemplified in Figure 9, field flow fractionation allows for a confirmation of the integrity of the self-assembled objects, since it is often coupled to orthogonal detection techniques, such as refractive index (RI) or light scattering measurements and absorption spectroscopy. Even if the application is PDT, this step is general and is performed without the PS inside the vector [29]. This enables a classification of the ability of the vectors to resist FBS with time. The comparison of PEO-PCL, PEO-PLA and PEO-PStyrene exhibiting similar sizes showed that PEO-PStyrene was the most stable. The advantage of PS for PDT is that their fluorescence will depend on their environment. This has been used to track existing transfer from the vector to albumin [145].

#### PS/vector affinity and loading

**Figure 10:**
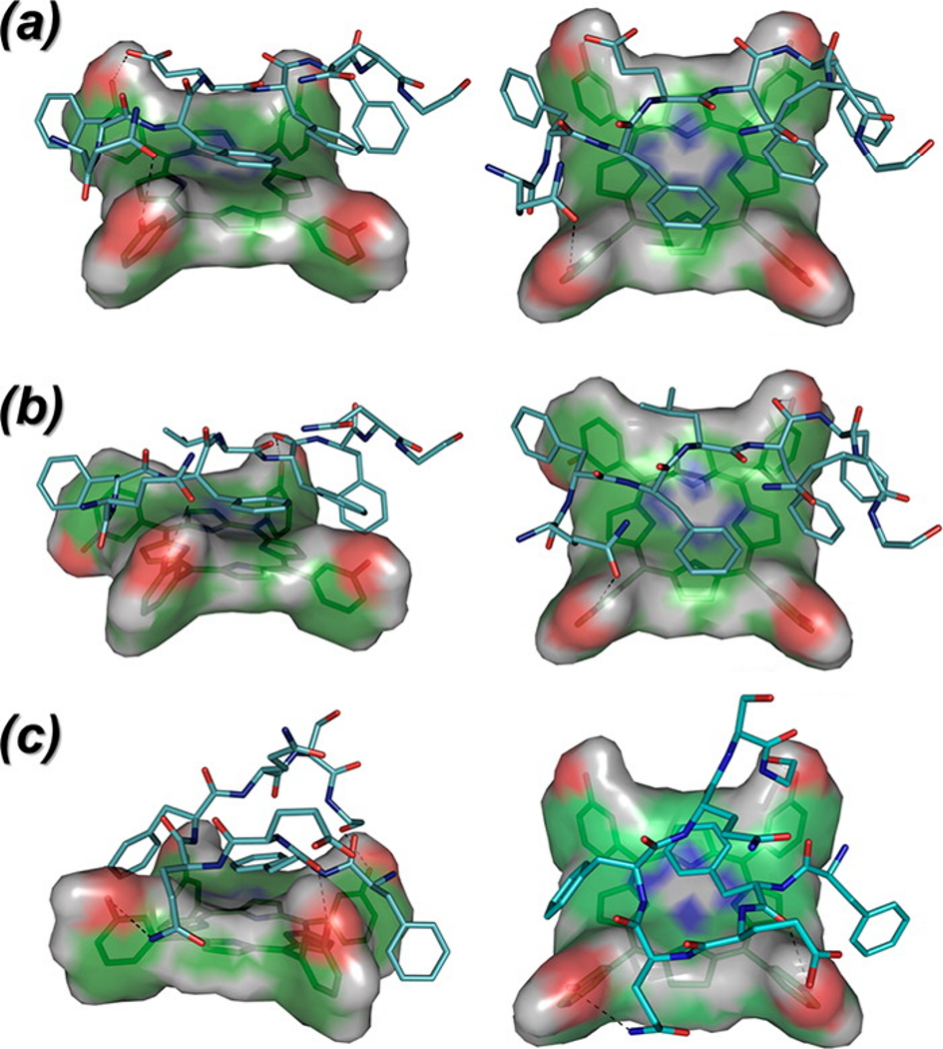
Idealized docking of 5,10,15,20-tetrakis(3-hydroxyphenyl)chlorin (*m-*THPC, shown as van der Waals surface) binding to peptide host sequences. Reprinted with permission from [145], copyright 2013 American Chemical Society.

The encapsulation of the PS by non-covalent binding implies that the choice of PS/vector pair is essential for the application. A comparison of the affinity between different PSs and pluronics polymers has been recently published and indicated differences in aggregation among all systems [32]. Going further, the PS might be specifically modified to optimize its affinity towards the desired vector. This has been performed on indocyanine in order to have good transport properties by HAS [131]. Once the PS has been chosen, the PS/vector affinity can be tuned by adjusting the polymer structure, introducing additional functional groups that are able to interact with the PS. This approach by itself is not new and Kataoka et al. used this strategy in 2005 to improve the loading of paclitaxel in PEO-PAsp micelles [146]. A typical recent example describes the introduction of benzylglycidyl ether groups on the hydrophobic backbone of polylactide, in order to improve the loading of aluminium phthalocyanine AlClPc [45]. Using a peptide backbone is another elegant and powerful means to optimize the affinity by adapting the amino acid sequence [145], as shown in Figure 10. Porphyrins have also been used in the polymer backbone to increase subsequent PS encapsulation [94]. Another approach to modulate the PS/vector affinity is the use of supramolecular complexes, particularly based on cyclodextrins. Several recent examples have been published [52–54,147] as well as a recent review [21].

**Figure 11:**
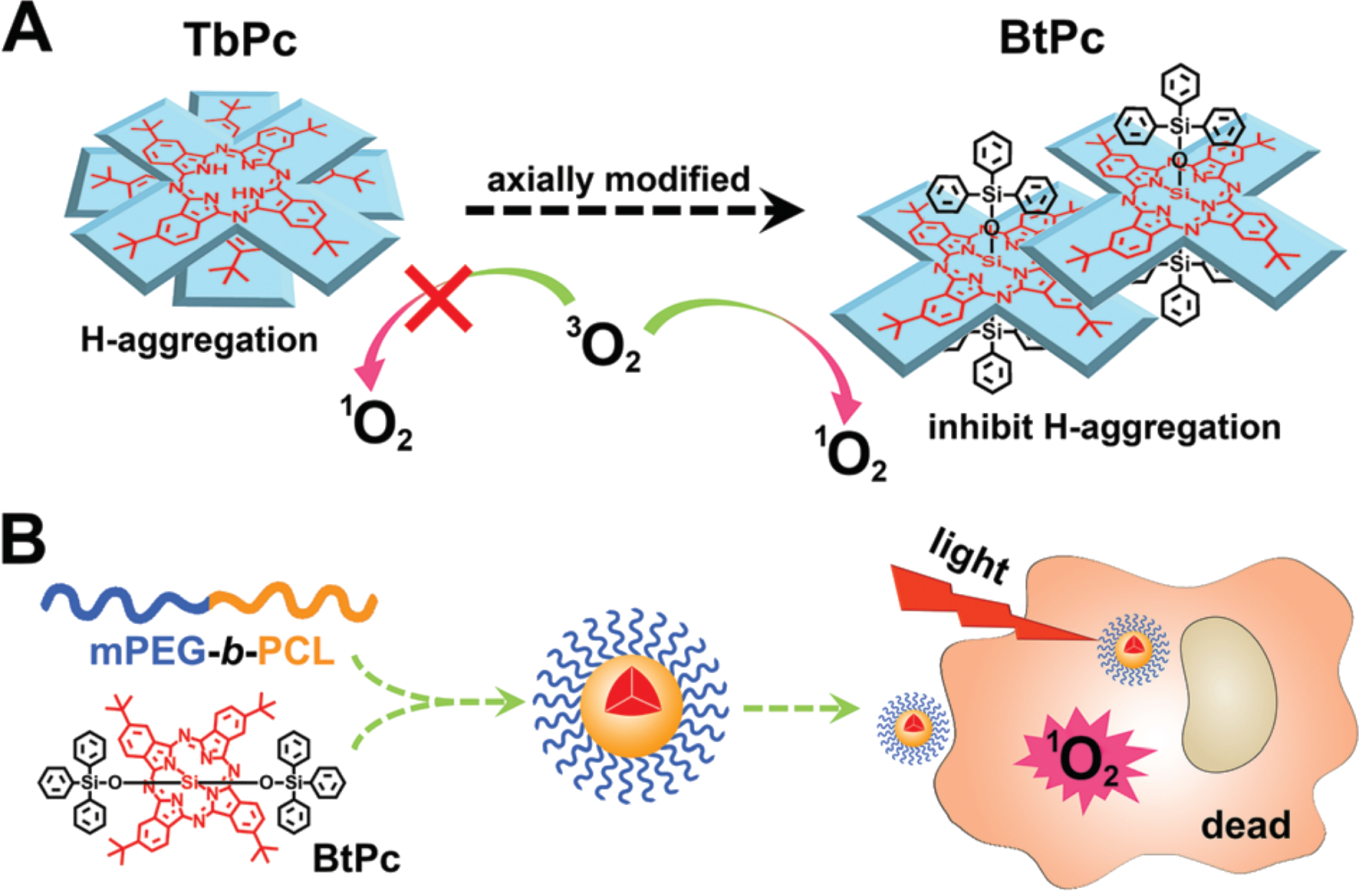
Modulation of PDT efficiency through introduction of bulky substituents on the PS, which inhibit aggregation. Reproduced with permission from [37], copyright 2018 The Royal Society of Chemistry.

The PDT efficiency strongly depends on the PS loading but a too high encapsulation might lead to aggregation of the PS inside the vector. This would decrease the interest of using the polymeric vectors. Therefore, the optimal loading has to be determined for each system. Ping et al. described a simple UV–visible spectroscopy method to assess the degree of aggregation inside the vector, thanks to the evolution of the PS spectrum (zinc phthalocyanine in their case) [148]. Shi et al. described the modification of the PS by introducing bulky aromatic ligands which inhibited the formation of H-aggregates [37] (Figure 11).

A special case is that of PICs, which are based on electrostatic interactions between a charged PS and an oppositely charged copolymer. This approach was first described for PDT by Kataoka et al. in 2005 [47] and has been examined from time to time since then. Recent examples [46,103] described the formation of PICs based on polylysine/tetrasulfonate phthalocyanine or heparin/polyethylene imine interaction. The approach proposed by Huh et al. is original since the polyelectrolyte complex is formed by association of a pheophorbide-modified heparin to polyethylene imine-β-carotene, the carotene moiety acting as a ROS scavenger as long as the PIC is formed [103]. The principle is that, once internalized, heparin will be degraded enzymatically and the PIC thus dissociates, enabling the production of ROS upon irradiation.

#### Stimuli-responsive vectors

This strategy has been examined a lot during the last years, in a general manner for nanomedicine but also for PDT. As already explained above, the principle is to benefit from the biological medium environment to trigger the drug release (the PS for PDT). The decrease in pH value in cancer tissues has been regularly used in nanomedicine to break a pH-sensitive bond leading to the dissociation of the vector and the subsequent release of the drug [80]. Recently, several studies focused on hypoxia, which might be considered as a strong drawback for PDT but could be reversed towards an asset if the vector can be rendered sensitive to this. The study published by Zhao from 2018 is a typical example [75]. The vector consisted of PEO and PAsp (modified with imidazole moieties) blocks linked via an azobenzene group. Because this group was shown to be cleaved by azoreductase under hypoxic conditions [149], the observed stronger cellular penetration [75] was explained by a de-PEGylation of the vector upon contact with the hypoxic tissues.

#### PS positioning

Since PDT relies on the local production of ROS to kill the diseased cells and since the lifetime of these ROS can vary from 0.01 to 0.18 µs [150–151] depending on their structure and environment, the location of ROS production is essential. For instance, singlet oxygen is known to travel only a few nanometers in aqueous solution [151]. It has been shown to be able to exit 20 nm polymeric micelles before being scavenged in a model solution [152]. However, the situation clearly changes if the vector is larger or if the PS has been released from its vector by the biological environment. That is why several studies focused on this point, examining either covalent or non-covalent systems. An elegant study [82] synthesized PEO-poly(benzylglutamate) PBLG copolymers, introducing an aza-BodiPy PS either at the end of PBLG (in the center of the vector) or at the PEO–PBLG junction (therefore at the border between hydrophilic and hydrophobic areas). In both cases, 90 nm vesicles were formed, but the PDT effect was increased in the latter case, when the PS was located at the PEG and PBLG junction.

For non-covalent systems, the problem of PS positioning has also been evaluated by several teams. Wilk’s team published an elegant characterization of PEO-PLA micelles encapsulating three different phthalocyanines, i.e., ZnPc, ZnPcF and tetrasulfonate-ZnPc [38]. They characterized PS positioning in solution by ^1^H NMR NOE and in the dried state by XPS coupled with ion sputtering, which enabled them to obtain depth profiles of the Zn atom. They corroborated a decreased ROS production of ZnPc to its location in the core of the vector, contrary to the two other PS, which were preferably distributed in the PEO corona. This means that, for cases where there is no PS release before PDT activation, the optimized positioning of the PS should be ideally near the hydrophilic/hydrophobic junction to limit the distance to be travelled by ROS and the possible early PS release.

The preceding paragraphs have examined each parameter in an independent manner. As already shown in Figure 7, interdependency clearly exists and the obtained therapeutic efficiency is a global result of all these parameters. In order to optimize a vector formulation, more systematic methods exist, namely the multivariate design of experiments (DOE) and the approach using Hansen solubility parameters (HSP).

DOE, in contrast to the usual one-variable-at-a-time (OVAT) method, generates experiments with multiple variables changing simultaneously. The subsequent mathematical treatment enables the optimization of experimental conditions to get the desired result and also indicates which parameters lead to synergistic or antagonist effects. This approach has been regularly used for the formulation of lipidic vectors [153] and is also increasingly assessed for polymeric systems [154–157]. To our knowledge, only two examples have performed DOE for PDT vectors. Both of them dealt with pluronic-based nanocarriers formulated with aluminium chloride phthalocyanine [34] or with hypericin for oncology and antimicrobial applications [33].

**Figure 12:**
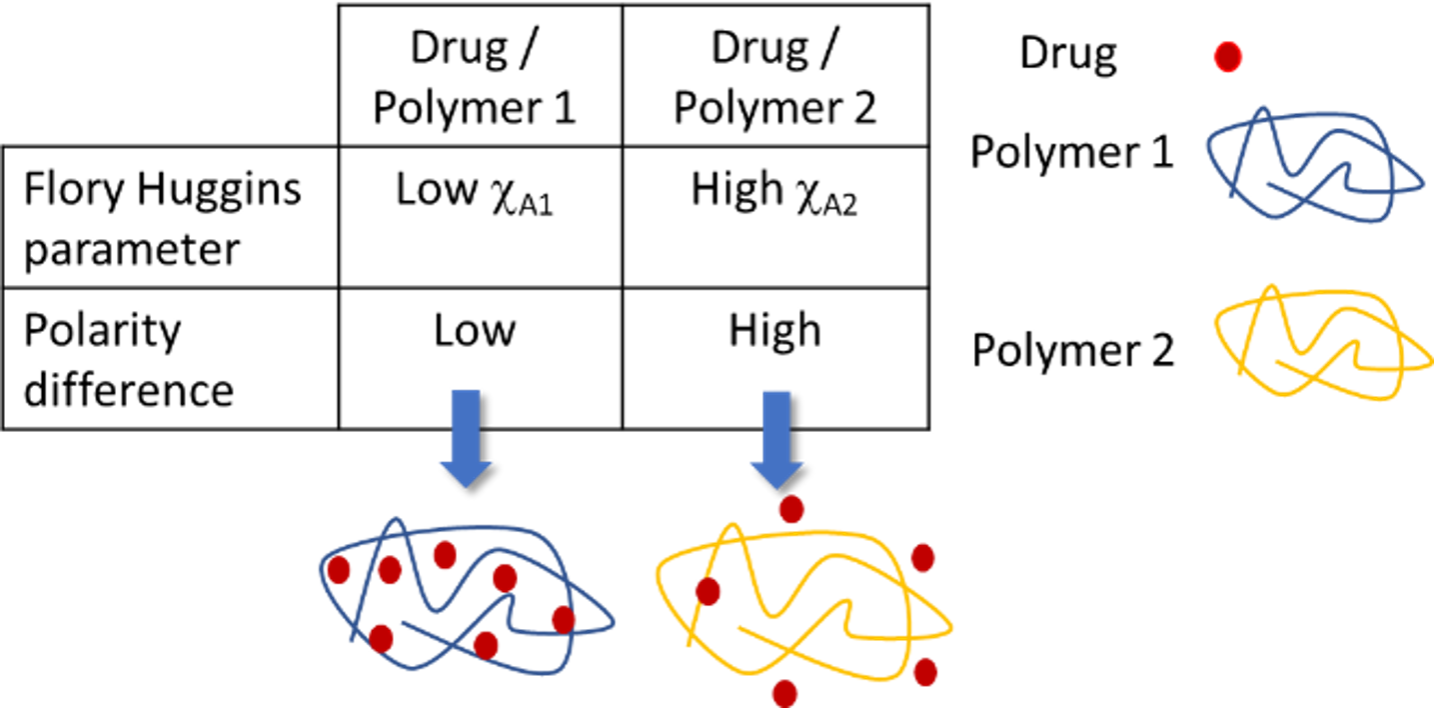
Use of Hansen solubility parameters to optimize polymeric nanovectors.

Whereas DOE does not make any assumption on the quality of the drug–vector affinity, the HSP method is based on the comparison of solubility parameters for both components. The principle, described in Figure 12, is that products exhibiting similar solubility will be more easily mixed. Recent examples for the formulation of polymeric vectors can be found in [134,158].

### Processes of interaction with membranes

When looking at the efficiency of a block copolymer-based nanocarrier, it is crucial to understand how it can interact with the cell membrane. The plasma membrane of eukaryotic cells is a highly selective barrier that protects all living cells from the surrounding microenvironment and efficiently limits the entry and exit of biomolecules and ions. Thus, nanovectors developed in the field of drug delivery have to overcome this physical barrier to penetrate within the cells. Understanding how nanoobjects and cell membranes interact is crucial but is clearly not trivial given the wide variety of nanoparticles properties (such as size, charge, shape, surface modification) and the complexity of biological systems. Interestingly, interactions of carbon-based and metallic nanoparticles with membranes and cellular uptake have been widely described [159–160], offering lines of thought in the case of cellular uptake of polymeric nanovectors that have been only little studied until now.

In the following paragraphs we will describe some recent efforts to understand the mechanisms involved in the internalization of self-assemblies into cells. We will first describe the use of model membranes, lipid self-assemblies the size, shape and composition of which can be controlled. Even if almost all the reported examples in this section deal with copolymers without the presence of a photosensitizer, the experimental methods and the results obtained are valid in the case of PDT when the photosensitizer is inside a nanocarrier. Besides, the photosensitizer could have non-negligible effects on the final physicochemical properties of the nanocarriers and the membranes. The examples reported here could inspire research in this sense in the field of PDT and we will describe some works on cells.

#### Interactions with model membranes

Lipid monolayers are a very simple but powerful tool to probe the interactions between a membrane and external compounds such as polymers. Using a monolayer made of 1,2-dipalmitoyl-*sn*-glycero-3-phosphocholine (DPPC) and cholesterol, Sandez-Macho et al. [161] have been able to show how different PEO-PPO-PEO copolymers with different sizes of the PEO blocks interacted with the membrane. They showed that the shorter the PEO blocks, the more the polymer expanded the surface area per lipid and increased the membrane permeability. These effects have been confirmed by haemolysis assays. Yaroslavov et al. [162] used DPPC/DOPG (1,2-dioleyl-*sn*-glycero-3-phosphoglycerol) monolayers to characterize the effect of various polybetaines on the membrane. They showed that upon complexation with the anionic monolayer, the polybetaine generated an expansion of the monolayer depending on the length of the spacer between the positive and negative charges of the betaine. The polybetaine generating the smaller expansion of the monolayer was also the one showing the least cytotoxicity on human breast carcinoma cell MCF7. Schwieger et al. [163] used monolayers made of different types of lipids, changing the nature of the polar heads or of the chains, to investigate the interactions between the lipids and two types of PGMA-PPO-PGMA triblock copolymers, one presenting fluorinated end chains. They showed that the fluorinated polymer incorporated more strongly in the monolayer than the non-fluorinated one. They noted that the strength of the interaction between the hydrophilic PGMA and the polar head depended on its nature and that it was stronger for phosphatidyl ethanolamine (PE) polar heads than for phosphatidyl choline (PC) polar heads. This effect was more apparent for the fluorinated polymer. Their experiments also suggested a partial miscibility of polymer in the lipid bilayer, forming some hybrid polymer–lipid monolayers.

Supported lipid bilayers are a type of planar model membrane where a complete bilayer is deposited on a substrate. This geometry allows for the use of classical techniques of surface analysis, such as microscopy or spectroscopy. Ramadurai et al. [164] used a 1,2-dioleyl-*sn*-glycero-3-phosphocholine (DOPC) bilayer deposited on a PDMS surface presenting microcavities to investigate the interactions between the membrane and different types of amphiphilic invertible polymers (AIP) micelles, a class of stimuli-responsive polymers that form micellar structures in polar solvents that can invert in non-polar solvents. Through fluorescence lifetime correlation spectroscopy, they showed that the most hydrophobic polymer they studied led to an increase of the membrane viscosity, attributed to the adsorption of the micelles on the bilayer. They also used electrochemical impedance spectroscopy and noted that the same polymer led to a strong decrease of the membrane resistance, linked to an increase of its permeability.

Liposomes are vesicles composed of a lipid bilayer. They have a close resemblance to cell membranes and can be used as substitute to investigate the interactions between cell membranes and nanocarriers. Liposomes can be produced in ways that allow for the control of the composition of the bilayer and of the internal aqueous phase. It is possible to prepare liposomes containing a self-quenching fluorescent dye in its internal aqueous phase and to follow the release of this dye under different types of stimuli. Because the dye release rate is related to the permeability of the liposome, this type of experiments is used to monitor the effects of polymers or nanoparticles on the integrity of membranes. Wilkosz et al. [165] used calcein-loaded 1-palmitoyl-2-oleoyl-*sn*-glycero-3-phosphocholine (POPC) liposomes to investigate the effect of cationic polymers and copolymers on the membrane. They showed that the densely substituted polycations generated a quick release of the calcein. They assumed that this was due to the formation of pores in the membrane. This theory was confirmed using molecular dynamics (MD) simulations. Palominos et al. [166] have prepared calcein-loaded DPPC liposomes mixed with two types of PCL-PEO-PCL copolymers of different block sizes. They showed that, in the concentration range that they studied, both polymers reduced the permeability of the membrane with the longer one having a greater effect. These results come as a confirmation of what they measured using two fluorescent dyes, laurdan and 1,6-diphenyl-1,3,5-hexatriene (DPH). By measuring the fluorescence polarisation of laurdan and the fluorescence anisotropy of DPH, it is possible to determine the physicochemical properties of the bilayer. Laurdan and DPH can “sense” their environment and insert themselves, respectively, at the interface of the bilayer and in the midst of the hydrophobic chains. Using this, they showed that the shorter copolymer had an effect only on the inner part of the bilayer while the longer one had an effect on both the interface and the inner part of the bilayer. By mixing POPC unilamellar liposomes with PEO-PPO copolymers of different block sizes and analyzing these mixtures with pulsed-field-gradient NMR, Zhang et al. [167] quantified polymer diffusion in the absence and presence of liposomes. From their results, they could assess the binding of the polymers to the liposomes. They showed that larger molecular weight and higher hydrophobicity of the polymer resulted in a higher binding percentage and liposomes surface coverage. They also noted that the binding percentage was independent of the incubation time, meaning that the polymer–membrane interactions occur immediately after mixing and reach an equilibrium state quickly. A recent example on pheophorbide a-loaded micelles interacting with giant vesicles shows a synergy between the photosensitizer and the polymer. An extended production of internal vesicles, resembling endosomes, is observed in the synthetic giant unilamellar vesicles, after interaction of pheophorbide a-loaded copolymer nanocarriers [168]. This does not happen when the photosensitizer is not loaded in the nanocarriers. All the performed experiments indicate that intimate interactions of the nanocarriers with the model bilayer are key for successful delivery and lipid oxidation is necessary for this pathway.

#### Computer-simulated interactions

**Figure 13:**
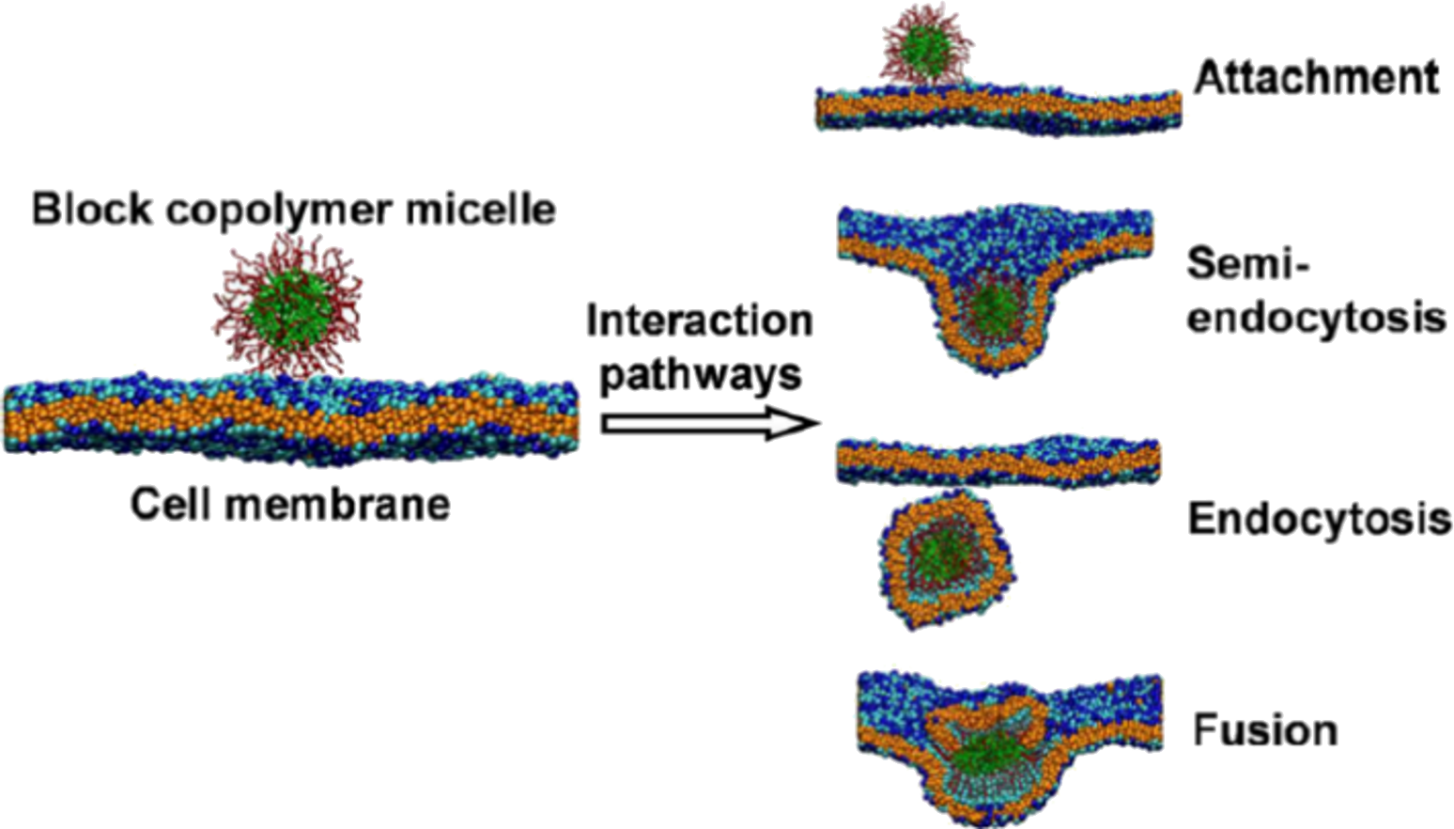
Types of pathways of block copolymer micelle–cell membrane interactions. Reprinted with permission from [172], copyright 2017 American Chemical Society.

Computer simulations enable one to model lipid bilayers and to examine how they behave in presence of polymer molecules or self-assemblies. Zaki and Carbone [169] used MD simulations to assess the effects of PEO-PPO-PEO triblock copolymers on DPPC bilayers under mechanical stress. They showed that the copolymer inserted itself in the membrane, leading to the formation of hybrid membrane with better mechanical properties. Houang et al. [170] compared the results obtained with MD simulations and with physiological studies. They used PEO-PPO copolymers with different PPO end groups and tested them as membrane stabilizer both in silico and in vitro. Using a POPC bilayer model under mechanical stress for their MD simulations, they showed that copolymers with a more hydrophobic end group could insert themselves deeper in the membrane bilayer while the more hydrophilic copolymers stayed close to the polar interface. These results validated the ones obtained from the animal model where the copolymer with the most hydrophobic end group was the one that helped muscle cells the most to resist mechanical stress. Raman et al. [171] used MD simulations to model a DOPC bilayer and to observe how PEO-PCL copolymers of different block sizes and their micelles could mix with the membrane. They showed that micelles with higher hydrophilic-to-hydrophobic ratio did not interact with the bilayer whereas those with lower hydrophilic-to-hydrophobic ratio were internalized over the course of their simulation. During this internalization, they saw a change in the structure of the micelles, going from a core–shell conformation to a Janus conformation with the PEO chains located at the interface close to the polar head groups and the PCL chains in the hydrophobic core of the bilayer. No effect on the area per lipid, average thickness and order parameter was measured. Guan et al. [172] explored different pathways of block copolymer micelles and membrane interactions by using a model of a lipid bilayer containing a proportion of lipid that could bind to the micelles. Figure 13 describes the results of their coarse-grained molecular simulations, where, by changing the binding strength, they isolated four types of pathways, i.e., attachment, semi-endocytosis, endocytosis, and fusion, linked to the wrapping parameter of the bilayer around the micelle. They showed that endocytosis was the most efficient pathway for the uptake of micelles and that fusion could result in membrane damage. They looked at the effects of the aggregation number of the micelles, length of the polymer and stiffness of the hydrophobic chains on the uptake of the micelles. Their results indicated that smaller aggregation number and polymer length led to a weaker uptake but higher values of these parameters generated more damage. They noted that lower hydrophobic chains stiffness could lead to micelles with a higher internalization efficiency and a lower toxicity.

#### Interactions with cells

Classical strategies to decipher cellular uptake mechanisms are based on selective chemical inhibition of the various endocytic processes or incubation at 4 °C instead of 37 °C to decrease cellular processes. Special attention should be paid to the use of fluorescent probes encapsulated within nanovectors to determine the fate of photosensitizers. Indeed, uncontrolled release of the fluorophore from the carrier may produce unreliable results. Conventional tools used to visualize/quantify cellular uptake are flow cytometry and confocal microscopy, which has been punctually combined with surface-enhanced infrared absorption spectroscopy (SEIRAS) [173] or FRET imaging [174]. In the context of PDT with polymeric nanoparticles, we identified some experimental qualitative/quantitative studies of interactions between nanoparticles and cell membranes, cellular uptake and drug release. Kerdous et al. proposed an original way to study the mechanisms of release of pheophorbide a-loaded in PEO-PCL, when exposed to human breast cancer cells MCF-7 [175]. Using a fluorescent confocal microscope setup that enabled concomitant spectroscopic and excited state lifetime measurements of the fluorescence emission signal of the photosensitizer, they demonstrated that pheophorbide delivery in a very minor way involved the internalization of nanoparticles. The major drug delivery mechanisms came from a direct transfer of the amphiphilic drug from the nanoparticle to the cell membrane by collision. Similarly, using PEO-PCL micelles loaded with fluorescent photosensitizer pheophorbide a or fluorescent copolymers, Till et al. demonstrated that pheophorbide a directly migrates from the micelles to the cell membrane without disruption of the vector or partial drug release in the vicinity of the cell [176]. At a different scale, Xue et al. observed by flow cytometry experiments after 4 h and 24 h of incubation with PEO-terminated ZnTPPC6-based poly disulfide ester (PEO-*b*-PTPPDS-*b*-PEO) micelles (134 nm) with A549 tumor cells, that the intracellular uptake of these polymeric micelles was a time-dependent process and proposed that cellular uptake came from endocytosis rather than the simple passive diffusion of free porphyrin with prolonged incubation time [88]. Wan et al. performed a detailed study about uptake mechanisms of 45 nm micelles of poly(aspartic acid)-*graft*-poly(ethylene oxide-indocyanine green) loaded with the antitumor drug paclitaxel, written PTX@PAsp-*g*-(PEO-ICG) [177]. Human ovarian cancer cells SK-OV-3 were pre-treated for 30 min with PBS at 37 °C as control and different inhibitory solutions, i.e., PBS at 4 °C for low-temperature incubation with slowed cellular processes, 2-deoxy-ᴅ-glucose (50 mM)/NaN_3_ (10 mM) to deplete the cells from energy (ATP) essential for endocytosis, or sucrose at 0.45 M as hypertonic solution to inhibit of clathrin-mediated endocytosis. After these pre-treatments, cells were incubated with polymeric micelles for 2 h at 37 °C before analysis by flow cytometry. While ATP depletion and hypertonic treatment failed to inhibit the cellular uptake of PTX@PAsp-*g*-(PEO-ICG), incubation at 4 °C reduced it by 66%. These results indicate that the cell uptake mechanism of PTX@PAsp-*g*-(PEO-ICG) was not through endocytosis (ATP required), but largely attributed to passive transportation. In conclusion, it has to be underlined that nanoparticles entering the cell via endocytic pathways will be directed to endosomal/lysosomal compartments, trapped within vesicles, while those entered through passive diffusion freely access the cytoplasm. In the latter case, depending on the photosensitizer (or drug)/polymer couple, distinct drug release mechanisms can be considered. These are photosensitizer release in the vicinity of cell membrane, direct transfer of the photosensitizer upon contact of the vector with the cell membrane, or penetration of the photosensitizer together with its carrier. Strategies developed to target specific subcellular organelles in the context of PDT will be discussed below. Finally, it has to be kept in mind that in silico and in vitro experiments on human cells represent a first step in understanding the interactions between membranes and polymeric nanovectors, meaning that in vivo experiments will be further needed to confirm cellular uptake efficacy.

### Subcellular organelle-targeted photodynamic therapy with polymeric nanovectors

The presence of photosensitizer and O_2_ under light irradiation during PDT generates reactive oxygen species (ROS) such as singlet oxygen (^1^O_2_), which is highly reactive and irreversibly oxidizes adjacent biological substrates such as signaling proteins or nucleic acids. Cell and tissue exposure to ^1^O_2_ results in the breakdown of cellular microstructures and cell death. The ^1^O_2_ lifetime was measured in vivo in rats after irradiation of aluminium tetrasulfonated phthalocyanine and appears to be 0.03–0.17 µs in liver and 0.04–0.18 µs in skin [150]. In vitro, the intracellular radius of action of ^1^O_2_ was estimated between 10 to 20 nm, corresponding to a lifetime of 0.01–0.04 µs [151]. Because of the fast decay and degradation of ^1^O_2_, photosensitizers have to be localized as close as possible to the targeted cellular organelles, mainly mitochondria, lysosome, or nucleus.

Challenges are to develop smart release approaches with precise spatiotemporal control for cancer therapy. Strategies adopted for intracellular targeting can be divided into passive, active and activable, in the latter case nanovectors remain photodynamically inactive until they reach the tumor site and more precisely the targeted intracellular compartment [178]. As already discussed in the section on stimuli-responsive polymers, providing nanovectors responding to an endogenous stimulus in addition to an external trigger can clearly improve the spatiotemporal control of their functions while limiting side effects from their inherent distribution in both normal and tumor tissues. Subcellular localization of the photosensitizer is largely governed by its concentration and its physicochemical properties (molecular weight, lipophilicity, amphiphilicity, ionic charge, and protein binding characteristics) [179]. On the one hand, lipophilic, anionic dyes generally localize in membrane structures (including plasma, mitochondrial, endoplasmic reticulum and nuclear membranes), while hydrophilic materials seem to accumulate in lysosomes [180]. On the other hand, cationic sensitizers such as rhodamines and cyanines preferentially accumulate in mitochondria [181] due to electrical potential gradients across the mitochondrial membrane, allowing a targeted approach for PDT [182–183]. Even if beyond the scope of this review, the physicochemical properties of the chosen photosensitizer also help to passively target intracellular compartments.

**Figure 14:**
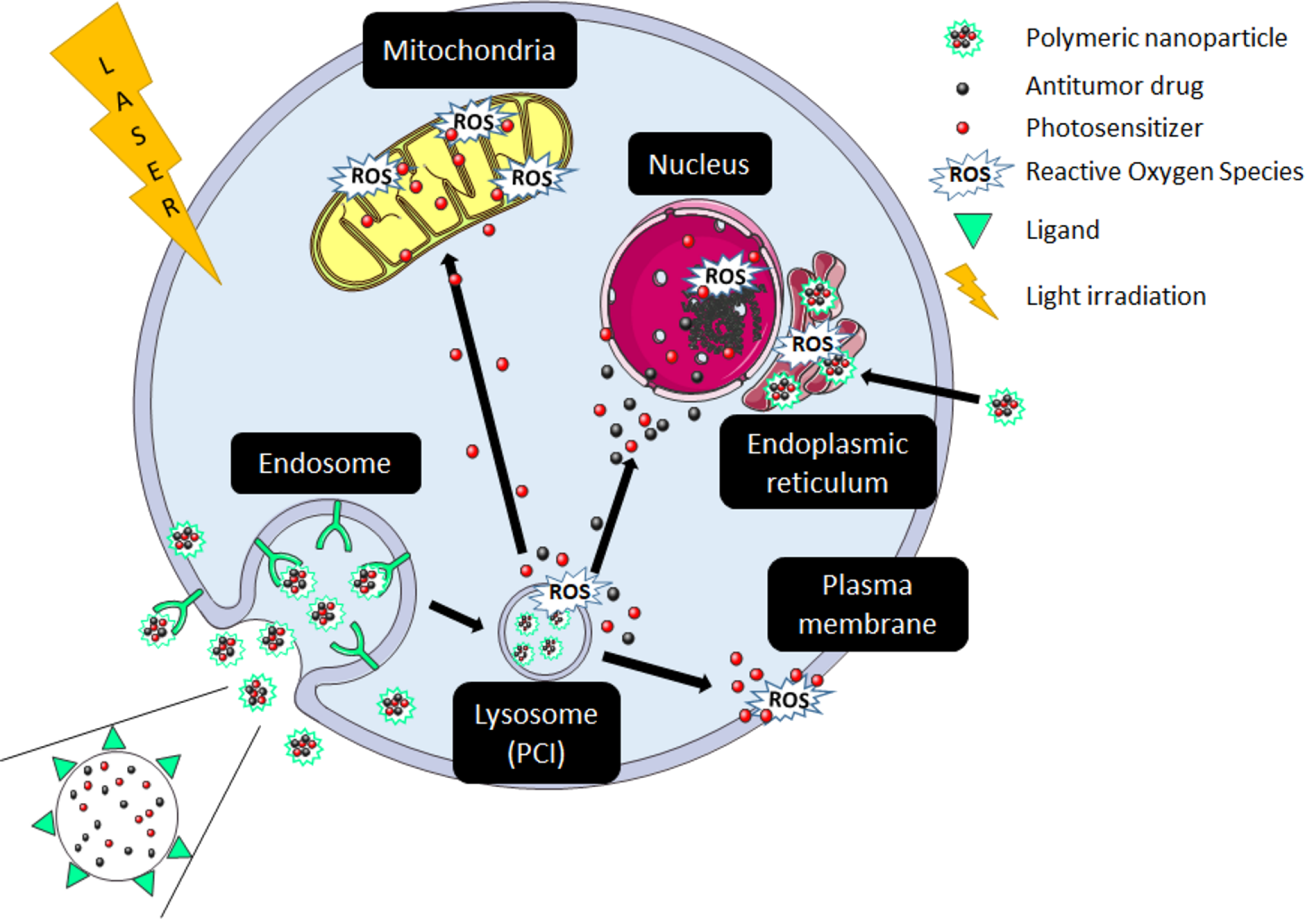
Schematic view of photodynamic therapy (PDT) strategies with polymeric nanovectors targeting subcellular organelles. PCI: photochemical internalization. ROS: reactive oxygen species.

Until today, several strategies for targeting subcellular organelles, including cell nucleus [184–185], lysosome [186], mitochondria, endoplasmic reticulum, and even plasma membrane, have been proposed to maximize the antitumor effects of PDT [187]. These strategies are schematized in Figure 14, listed in Table 4 and presented below.

#### Endosome/lysosome-targeted PDT and photochemical internalization

An approach described to target lysosomes was to synthetize positively charged ruthenium(II) polypyridyl complexes that selectively localize in lysosomes through endocytosis and induce serious phototoxicity in human 3D tumor spheroids after two-photon photodynamic therapy [186]. Another promising way to develop lysosome-targeted PDT relies on photochemical internalization (PCI) strategies based on polymeric nanovectors. PCI is a concept designed by Berg et al. based on photodynamic therapy [188–189]. This technique results from the activation of a photosensitizer at a specific wavelength after its cellular internalization in endosomes. The photosensitizer in the excited state induces the formation of reactive oxygen species such as singlet oxygen that will disrupt the endosomal membrane. This technique allows for a spatiotemporal control of the release of the endosomal content into the cytosol [190]. PCI is applied for the codelivery of a photosensitizer with an active agent such as nucleic acids [191], proteins [192], anticancer agents [193–195], or nanoparticles [48,196]. In the area of nanomedicine, this concept may be a powerful tool when associated with nanovectors such as copolymer micelles to increase the efficacy of the drug delivery [168]. This strategy was particularly successful in the case of drug delivery of camptothecin using dual degradable and pH-sensitive nanoparticles of PEO-acetal [72] and hematoporphyrin as a photosensitizer or of doxorubicin encapsulated in block copolymer micelles of porphyrin-modified PEO-PCL [79].

#### Plasma membrane-targeted PDT

Usually, PDT efficacy requires the adequate cellular uptake of polymeric vectors loaded with photosensitizer. Since the cell plasma membrane plays the fundamental role of a selective barrier leading frequently to an inadequate internalization of nanovectors, some authors proposed to target the plasma membrane integrity through the peroxidation of lipids via ROS produced during PDT in order to eradicate tumor cells [197]. Interestingly, the loss of plasma membrane integrity because of PDT leads to cell necrosis rather than apoptosis [198] further eliciting antitumor immune responses. Distinct strategies were proposed to target the plasma membrane. Among them, Kim et al. developed ZnPc-loaded membrane fusogenic liposomes, engineered to fuse with the plasma membrane and deliver ZnPc within it [199]. They confirmed that membrane localization of ZnPc molecules led to rapid membrane disruption upon irradiation and to a subsequent necrosis-like cell death. Recently, Jia et al. developed self-assembled polymeric nanoparticles (80 nm diameter) composed of PEO and glycol chitosan (GC) and loaded with protoporphyrin IX (GC-PEO-PpIX) [200]. Interestingly, the PpIX moieties exhibit a high affinity for plasma membrane. Indeed, when the nanoparticles encountered the plasma membrane, the nanoparticles dissassembled and PpIX photosensitizer remained anchored to the lipid bilayer through multisite anchoring. It was observed in vitro on A549 human pulmonary cancer cells that plasma membrane targeted-PDT acted in two synergistic ways: plasma membrane integrity was first lost, leading secondly to a massive entry of nanovectors within the cell, causing the destruction of intracellular organelles. PpIX presented in vitro a remarkable PDT efficacy when encapsulated within GC-PEO-PpIX micelles compared to free PpIX (i.e., after laser irradiation at 635 nm at 14 mW·cm^−2^ for 1 min, cell viability was respectively 50% and more than 95%). In vivo results on nude mice bearing U14 subcutaneous tumors demonstrated that GC-PEO-PpIX micelles achieved a good tumor accumulation and retention, paving the way to use them as theranostic agent for image-guided PDT. Mice treated with GC-PEO-PpIX had the tumors eliminated without regrowth within 22 days of observation, while free PpIX exhibited little therapeutic efficacy. In conclusion, the authors demonstrated that plasma membrane-targeted PDT efficiently induced plasma membrane permeability upon laser irradiation, allowing for a massive penetration of nanovectors loaded with photosensitizer within the cytoplasm. These synergic processes promise to bypass improper cellular uptake or lysosomal escape issues faced by therapeutic strategies based on nanovectors. It could be a promising solution to avoid/limit cancer cell resistance to drugs observed with conventional antitumor therapies.

#### Mitochondria-targeted PDT

Mitochondria are a target of choice because they are numerous in cells, widely distributed in the cytoplasm, and play a pivotal role in metabolism and cell apoptosis [201]. Furthermore, molecular oxygen, which is a pre-requisite for PDT efficacy, is present in mitochondria because it is required as a terminal electron acceptor for ATP production [202]. Some authors chose to chemically modify known photosensitizers such as pheophorbide a with carboxybutyltriphenylphosphonium to specially target mitochondria [203]. TPP-based lipophilic cations have the ability to cross the mitochondrial membrane. By combining this therapeutic agent with folate-cholesteryl albumin (FA-chol-BSA), they obtained nanoparticles of 161.4 ± 14.3 nm of diameter which were readily taken up by murine and human tumor cells in vitro. Interestingly, the modified photosensitizer specifically accumulated within the mitochondria, leading to mitochondrial dysfunction and cell apoptosis after light irradiation. Nanoparticles loaded with TPP-Pheo a were quicker to induce an antitumor effect in vivo in mice model than non-modified Pheo a, which did not target mitochondria. Another strategy proposed was to add the lipophilic TPP cation directly on polymers used to produce nanoparticles, instead of modifying a photosensitizer [204–205]. This approach was followed with FDA-approved and biodegradable poly(lactic-*co*-glycolic acid) PLGA nanoparticles loaded with the antitumor drugs lonidamine and α-tocopheryl succinate. A higher mitochondrial uptake of the chemotherapeutics was demonstrated when nanoparticles were targeting mitochondria thanks to TPP. Nanoparticles are classically taken up by the endosomal pathway, which represents a physical barrier for mitochondria-targeted nanoparticles. But interestingly, these PLGA-*block*-PEO-TPP nanoparticles displayed amazing endosomal and lysosomal escape properties. The authors proposed that positively charged PEG exhibits a buffering capacity preventing endosomes acidification. This increases ATPase activity and counter ions accumulation in endosomal vesicles leading to osmotic swelling, membrane disruption and nanoparticle release within the cytoplasm, in a similar mechanism as observed with the strongly buffering polyamines poly(ethylene imine) (PEI) or PAMAM [206]. Self-assembled PEG-PCL-TPP bromide micelles (40 nm diameter) efficiently deliver coenzyme Q10 antioxidant within mitochondria to restore cellular functions [207]. Other mitochondrial targeting strategies have been developed for PDT. Among these are hollow silica nanoparticles loaded with catalase enzyme to produce the O_2_/chlorin-e6 photosensitizer/pH-responsive anionic polymer PEG/2,3-dimethylmaleic anhydride-*co*-poly(allylamine hydrochloride)/(3-carboxypropyl)TPP bromide to target mitochondria [208], pyropheophorbide a loaded onto nanographene oxide (NGO) particles [209] because single-walled carbon nanotubes previously showed a tropism for mitochondria [210], and an iridium(III) complex (Ir-P(ph)_3_) [211]. Although they do not rely on polymeric nanoobjects, these approaches are important to note for a better overview over possibilities to target mitochondria.

#### Nucleus-targeted PDT

Passive diffusion through the nuclear pore complexes is a way to enter a cell nucleus. Gaus et al. demonstrated that passive nuclear targeting can be achieved by adapting polymeric nanoparticle shapes to the architecture of nuclear pore complexes [212]. Working with poly(oligoethylene glycol methacrylate)-*block*-poly(styrene-*co*-vinylbenzaldehyde) nanoparticles, they demonstrated that rod-like (5–10 nm × 100–300 nm) and worm-like nanoparticles (5–10 nm × 400–700 nm) were more suitable than micelles and vesicles to penetrate the cell nucleus and deliver the associated doxorubicin. Even if this work was not led in the context of PDT, it demonstrated that the shape of polymeric nanoparticles appears to be a relevant criterion to design nanovectors capable of passively diffuse across the nuclear membrane. An original work was realized by El-Akra et al*.* in order to target the nucleus of estrogen-dependent cancer and vascular endothelial cells to eliminate both tumor and blood vessel cells using PDT [213]. For this purpose, estradiol and pheophorbide a (E-Pheo a) were linked by two amide bonds via oxoethylene or oxopropylene spacers. Efficient cellular uptake and intranuclear localization was confirmed in vitro in human MCF-7 breast cancer cells known to highly express estrogen receptors (EsR). E-Pheo a was shown to be seven times more phototoxic than a control compound in EsR-positive MCF-7 cell lines and human EA.hy926 vascular endothelial cells. In EsR-negative SKBR3 cells the same phototoxicity was observed for both compounds. Some PDT strategies, although not using polymeric nanovectors, were designed to target the nucleus using a cyclometalated iridium(III) complex [214]. However, it must be kept in mind that nucleus-targeted PDT agents caused great damage to the DNA of cancer cells, which also generates a high risk of genetic mutation in surrounding healthy cells.

#### Endoplasmic reticulum-targeted PDT

**Figure 15:**
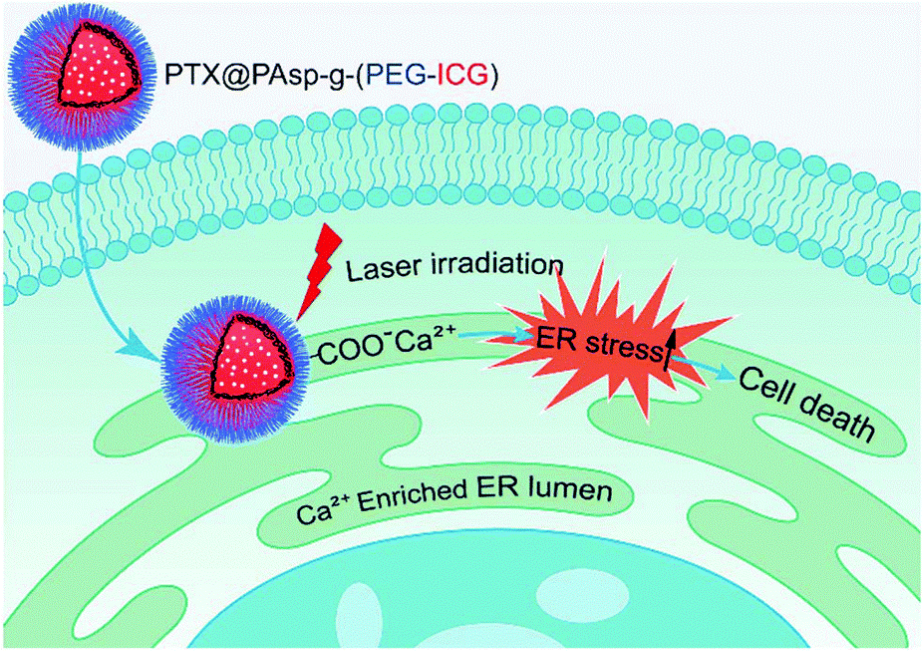
Illustration of the PTX@PAsp-*g*-(PEG-ICG) ER-targeting process and mechanism of cell death. PTX@PAsp-*g*-(PEG-ICG) micelles accumulate in the ER lumen through the coordination affinity of the Ca^2+^ ions to the carboxy groups of PAsp. Upon laser irradiation of the photosensitizer ICG, the generated ROS would lead to elevated stress and induce cancer cell death. ER: endoplasmic reticulum. ICG: indocyanine green. ROS: reactive oxygen species. Reproduced with permission from [177], copyright 2018 The Royal Society of Chemistry.

The endoplasmic reticulum (ER) is a dynamic organelle dedicated to protein synthesis and folding, calcium storage and lipid/carbohydrate metabolism [215]. Some authors proposed as therapeutic strategy to interfere with ER functions by generating stress through ER-targeted PDT. In vitro cellular assay was developed to screen ER-targeting photosensitizers with ideal photoactivity [216]. Wan et al. designed ER-targeted micelles for PDT that can be efficiently loaded with the antitumor drug paclitaxel [177]. It is a new type of biodegradable comb-like polymer, namely poly(aspartic acid)-*graft*-(PEG-ICG). ICG, in addition to being an imaging agent, exhibits PDT and photothermal therapy effects under near-infrared irradiation. The authors hypothesized that the carboxy-containing polymers are able to target the ER through the strong coordination affinity of Ca^2+^ ions to the carboxy groups of the polymer owing to the extremely high concentration of Ca^2+^ ions within the ER compared to the cytosol (Figure 15).

Using SK-OV-3 human ovary tumor cells, they demonstrated that micelles uptake mechanism was largely attributed to passive diffusion and not to endocytosis. Under laser irradiation (0.2 W cm^−2^, 785 nm, 30 s), PTX@PAsp-*g*-(PEG-ICG) micelles induced a ten-fold ROS production in SK-OV-3 cells, compared to the non-irradiated cells, underlining the high potential of these micelles for PDT. Cell death through apoptosis was measured in vitro in glioma cancer cells U-87 MG. While PTX@PAsp-*g*-(PEG-ICG) micelles without irradiation induced only 10.6% of cell apoptosis, cell apoptosis jumped to 73.2% after irradiation (0.2 W·cm^−2^, 785 nm, 5 min). The viability of U-87 MG cells was assessed in vitro after incubation for 24 h with PTX@PAsp-*g*-(PEG-ICG). Free paclitaxel at a concentration of 2.5 μg·mL^−1^ decreased cell viability by 40% while PTX@PAsp-*g*-(PEG-ICG) with laser irradiation (2 W·cm^−2^, 785 nm, 30 s) reduced the cell viability by 100%, indicating that PDT and drug vectorization remarkably enhanced chemotherapeutic effects. In vivo experiments on nude mice bearing U-87 MG tumor indicated that PTX@PAsp-*g*-(PEG-ICG) micelles preferentially accumulate in the tumor site. The mouse group “PTX@PAsp-*g*-(PEG-ICG) micelles treated with laser irradiation” displayed a more effective tumor inhibition, with complete tumor remission in two mice at day 21, than other groups (PBS control, taxol-treated, non-irradiated PTX@PAsp-*g*-(PEG-ICG) micelles). In this example, authors aimed and achieved to induce tumor cell death by causing stress through ER-targeted PDT. However, biologists are increasingly exploring the causes and consequences of ER stress in malignancy. Accumulation of misfolded proteins in the endoplasmic reticulum causes ER stress and activation of the unfolded protein response, which in turn promotes cancer development and progression through active modulation of immune cell functions [217]. Thus, nowadays, clinical antitumor therapeutic strategies aim to control ER stress responses in cancer cells to enhance the efficacy of standard chemotherapies instead of creating new ER stress [218].

**Table 4:** Overview of the subcellular organelle-targeted photodynamic therapy with polymeric nanovectors strategies. PS: photosensitizer. PM: plasma membrane.

targeted cellular compartment	targeting	stimuli	polymer	PS and associated drug	biological tests	ref

mitochondria	1: folic acid targeting endo/lysosomes 2: ammonium-functionalized cations (porphyrin) targeting mitochondria	1: low-pH-triggered lysosomal escape 2: redox-induced disassembly (cytoplasm) and subsequent drug release 3: light irradiation (mitochondria)	PEG-PDBO-BPT	5-(3-hydroxy-*p*-(4-trimethylammonium)butoxyphenyl)-10,15,20-triphenylporphyrin chlorin (MTPP) + camptothecin	in vitro + in vivo	[219]
	carboxybutyltriphenyl­phosphonium	light irradiation	folate-cholesteryl albumin (FA-chol-BSA)	carboxybutyltriphenyl­phosphonium-pheophor­bide a (TPP-Pheo a)	in vitro + in vivo	[203]
endosomes	folic acid	pH-sensitive	PEG-poly (β-benzyl-ʟ-aspartate)	pheophorbide a (hydrophobic)	in vitro	[220]
cytoplasm	folic acid targeting endo/lysosomes	light-triggered drug release through ROS production	mPEG-*b*-PPADT poly(1,4-phenyleneacetone dimethylene thioketal) (PPADT)-PEG	*meso*-tetraphenylporphyrin + paclitaxel	in vitro + in vivo	[221]
	biotin targeting endo/lysosomes	light-triggered drug release through ROS production	mPEG-*b*-PPADT poly(1,4-phenyleneacetone dimethylene thioketal) (PPADT)-PEG	silicon 2,3-naphthalocyanine bis(trihexylsilyloxide) (SiNc) + paclitaxel	in vitro + in vivo	[222]
plasma membrane	protoporphyrin IX moieties	light irradiation	glycol chitosan (GC) and polyethylene glycol (PEG)	protoporphyrin IX	in vitro + in vivo	[200]
endoplasmic reticulum	coordination affinity of the Ca^2+^ ion to the multi-carboxyl group of the polymer	light irradiation	poly(aspartic acid) and polyethylene glycol (PEG)	indocyanine green + paclitaxel	in vitro + in vivo	[177]

## Conclusion

In this review, we have described the developments of the last five years regarding block copolymer nanocarriers used in PDT. The main concerns have been the control of the copolymer structure, which must be designed in order to optimize the nanocarrier performance in term of photosensitizer loading and release at the tumor tissue. Sophisticated stimuli-responsive systems have been conceived to allow for drug release according to the environmental conditions. Some effort has also been addressed to overcome hypoxia, a hallmark of tumor tissues, and a main drawback for PDT. Interestingly, the main trend of the last three years (about 35% of the cited work published during this period) is aimed at developing intelligent “all in one” nanocarriers in which different drugs are loaded in the same nanocarrier. Combined treatments are more and more proposed and PDT is often associated with chemotherapy or photo-thermal therapy. Theranostic nanotechnologies, where diagnostic is combined with therapy, enable multimodal imaging (fluorescence or near-infrared fluorescence imaging, MRI, PET) thanks to the addition of fluorescent molecules, contrast agents or radioactive species. These unique, multifunctional nanocarriers are complex by nature and their mechanism of action is not well understood, as we stressed in the section on processes of interaction with membranes. Besides, another often overlooked aspect is the fate of polymer nanocarriers in biological fluids and in particular the possible influence of other macromolecules such as proteins on their stability and structure. This has been assessed in the field of inorganic nanomaterials, where more and more attention has been addressed towards the nano–bio interface in order to understand the interactions between engineered nanomaterials and biological systems. This established approach for inorganic nanoparticles is also essential in the case of soft self-assembled nanocarriers and should be more often examined. Indeed, the nanovector should not be considered by itself but characterized with its associated protein corona. This new assembly vector/corona should be defined as the real nanocarrier of the drug or the PS.

Some efforts have been also made in terms of cell targeting in order to improve the nanocarrier accumulation in the tumor tissues. Another aspect of targeting that is less taken into account in the nanocarrier community is the possibility to bring the photosensitizer to the organelles inside the cell.

We are convinced that the essential directions of future research should be the following:

1. It would be important to decipher the mechanism of nanovectors with a physicochemical perspective, i.e., to understand the main parameters in terms of copolymer nature and structure that favor photosensitizer cell internalization. To date, only theoretical works and some physicochemical studies address it mostly using biomimetic membranes. This aspect is essential to guide design choices to improve therapy efficiency.
2. The scientific community should make some efforts to establish standard PDT protocols so that the different proposed nanocarriers could be compared. The necessity for a “minimum information standard” [223] has been already proposed for inorganic nanomaterials. It consists in a standardization of material characterization, biological characterization and experimental details. This process would allow one to select the best nanocarrier properties thus sensibly helping the development of polymer nanocarriers and possibly resulting in more chances to go to clinical trials.
3. Finally, the design of nanocarrier targeting properties remains a challenge, going largely beyond the PDT application. Increasing knowledge about the protein corona will undoubtedly shed a new light on this topic.

## List of Abbreviations

AIE: aggregation induced emission; AIP: amphiphilic invertible polymers; DOE: design of experiments; DOPC: 1,2-dioleyl-*sn*-glycero-3-phosphocholine; DOPG: 1,2-dioleyl-*sn*-glycero-3-phosphoglycerol; DPPC: 1,2-dipalmitoyl-*sn*-glycero-3-phosphocholine; DPH: 1,6-diphenyl-1,3,5-hexatriene; EPR: enhanced permeation and retention effect; ER: endoplasmic reticulum; FA: folate; FBS: foetal bovine serum; FDA: Food and Drug Administration; FI: fluorescence imaging; FRET: Förster resonance energy transfer; GC: glycol chitosan; GSH: glutathione; HLB: hydrophilic–lipophilic balance; HSP: Hansen solubility parameter; HSA: human serum albumin; ICG: indocyanin green; MD: molecular dynamics; MRI: magnetic resonance imaging; NIR: near infra red; NIRFI: near infra red fluorescence imaging; NOE: nuclear Overhauser effect; OVAT: one-variable-at-a-time; PA: photoacoustic imaging; PAMAM: poly(amidoamine); PAsp: poly(aspartate); PBLG: poly(γ-benzyl-ʟ-glutamate); PBS: phosphate buffered saline; PCI: photochemical internalization; PCL: poly(ε-caprolactone); PIC: poly ion complexes; PDMS: poly(dimethyl siloxane); PDT: photodynamic therapy; PEG: poly(ethylene glycol); PEI: poly(ethylene imine); PEO: poly(ethylene oxide); PET: positron emission tomography; PFC: perfluorocarbon; PGMA: poly(glycidyl methacrylate); PLA: poly(lactic acid); PLGA: poly(lactic-*co*-glycolic acid); Plys: poly(lysine); PMAGP: poly(6-*O*-methacryloyl-ᴅ-galactopyranose); POEGMA: poly[oligo(ethylene glycol) methyl ether methacrylate]; POPC: 1-palmitoyl-2-oleoyl-*sn*-glycero-3-phosphocholine; PpIX: protoporphyrin IX; PPO: poly(propylene oxide); PTT: photothermal therapy; PTX: paclitaxel; PS: photosensitizer; ROS: reactive oxygen species; TCPP: 5,10,15,20-tetrakis(4-carboxyphenyl)porphyrin; TEM: transmission electron microscopy; TPETP: tetraphenylethenethiophene; TPP: triphenylphosphonium; ^1^H NMR: proton nuclear magnetic resonance; XPS: X-ray photoelectron spectroscopy; ZnPc: zinc phthalocyanine
